# Static Clathrin Assemblies at the Peripheral Vacuole—Plasma Membrane Interface of the Parasitic Protozoan *Giardia lamblia*

**DOI:** 10.1371/journal.ppat.1005756

**Published:** 2016-07-20

**Authors:** Jon Paulin Zumthor, Lenka Cernikova, Samuel Rout, Andres Kaech, Carmen Faso, Adrian B. Hehl

**Affiliations:** 1 Institute of Parasitology, University of Zurich, Zurich, Switzerland; 2 Center for Microscopy and Image Analysis, University of Zurich, Zurich, Switzerland; University of California Los Angeles, UNITED STATES

## Abstract

*Giardia lamblia* is a parasitic protozoan that infects a wide range of vertebrate hosts including humans. Trophozoites are non-invasive but associate tightly with the enterocyte surface of the small intestine. This narrow ecological specialization entailed extensive morphological and functional adaptations during host-parasite co-evolution, including a distinctly polarized array of endocytic organelles termed peripheral vacuoles (PVs), which are confined to the dorsal cortical region exposed to the gut lumen and are in close proximity to the plasma membrane (PM). Here, we investigated the molecular consequences of these adaptations on the *Giardia* endocytic machinery and membrane coat complexes. Despite the absence of canonical clathrin coated vesicles in electron microscopy, *Giardia* possesses conserved PV-associated clathrin heavy chain (*Gl*CHC), dynamin-related protein (*Gl*DRP), and assembly polypeptide complex 2 (AP2) subunits, suggesting a novel function for *Gl*CHC and its adaptors. We found that, in contrast to GFP-tagged AP2 subunits and DRP, CHC::GFP reporters have no detectable turnover in living cells, indicating fundamental differences in recruitment to the membrane and disassembly compared to previously characterized clathrin coats. Histochemical localization in electron tomography showed that these long-lived *Gl*CHC assemblies localized at distinctive approximations between the plasma and PV membrane. A detailed protein interactome of *Gl*CHC revealed all of the conserved factors in addition to novel or highly diverged proteins, including a putative clathrin light chain and lipid-binding proteins. Taken together, our data provide strong evidence for giardial CHC as a component of highly stable assemblies at PV-PM junctions that likely have a central role in organizing continuities between the PM and PV membranes for controlled sampling of the fluid environment. This suggests a novel function for CHC in *Giardia* and the extent of molecular remodeling of endocytosis in this species.

## Author Summary

In canonical clathrin mediated endocytosis (CME) models, the concerted action of ca. 50 proteins mediates the uptake of extracellular components. The key player in this process is clathrin which coats transport intermediates called clathrin coated vesicles (CCV). The intestinal parasite *Giardia lamblia* has undergone extensive remodeling during colonization of the mammalian duodenum. Here, we report on unique features of this parasite’s endocytic system, consisting of fixed peripheral vacuoles (PV) in close proximity to the exposed plasma membrane (PM), with no discernible CCVs. Using state-of-the-art imaging strategies, we show that the surface of *Giardia* trophozoites is pock-marked with PM invaginations reaching to the underlying PV membrane. Co-immunoprecipitation and analysis of protein dynamics reveal that, in line with the absence of CCVs, giardial clathrin assemblies have no dynamic behavior. CHC still remains associated to AP2 and dynamin, both conserved dynamic CME components, and to a newly identified putative clathrin light chain. The emerging model calls for giardial clathrin organized into static cores surrounded by dynamic interaction partners, and most likely involved in the regulation of fusion between the PM and the PVs in a “kiss-and-flush”-like mechanism. This suggests that Giardia harbors a conceptually novel function for clathrin in endocytosis, which might be a consequence of host-parasite co-evolution.

## Introduction

*Giardia lamblia* trophozoites proliferate in a very narrow ecological niche, attached to the epithelium of the upper small intestine of vertebrate hosts [1]. This high degree of specialization required significant host-parasite co-evolution-driven morphological adaptations. Notably, *Giardia* trophozoites show a distinctive dorso-ventral polarization with a novel organelle for direct physical attachment to the gut epithelium. Within the diplomonad phylum, *Giardia* is the only known genus, to have evolved this so called ventral disc (VD), a cytoskeleton structure shaped like a suction cup underneath the PM, on the flat ventral domain [2]. The cell body proper is dome-shaped and delimited by the PM on the dorsal side. This defines the overall cell morphology and converges with the ventral PM into a flat, tapered tail at the posterior end. Trophozoites are preferentially tightly attached to the gut epithelium or to artificial supports in cell culture systems, exposing the dorsal PM to the nutrient rich lumen. This strong dorso-ventral polarization is also reflected in the subcellular arrangement of organelles. To communicate with the fluid phase environment trophozoites use a distinctive set of endocytic organelles [3]. This endocytic system consists of a fixed non-anastomosing set of organelles arrayed exclusively in a cortical region underneath the dorsal PM and at the center of the VD, termed PVs [4]. The endo/lysosomal nature of PV organelles and their ability to sample the fluid phase environment is widely recognized [3], but the exact mode of endocytic internalization and further processing/transport of endocytosed substances towards the cell interior remains elusive. Moreover, there are several examples of exocytic cargo that have been localized to PVs [5–9] whilst a constitutive secretory pathway in trophozoites has not been fully characterized yet [8]. This suggests these organelles to be at the crossroads of endo- and exocytosis and therefore a direct bidirectional interface for host parasite interaction.

Clathrin mediated endocytosis (CME) is by far the best understood pathway of internalization in eukaryotic cells. Many of the approximately 50 factors involved in the selective uptake of plasma membrane subdomains via the formation of transport intermediates called clathrin coated vesicles (CCVs) have been functionally characterized [10]. The basic concept as well as structural and functional aspects of CCV formation are conserved from mammals to early diverged protozoa such as African trypanosomes; this is also reflected in the presence of clathrin heavy chain-encoding ORFs in all sequenced eukaryotic genomes but microsporidia [10].

The formation of a CCV can be divided into distinct steps each of which is amenable to documentation by fluorescence and/or electron microscopy (EM) [11–14]. In transmission EM sections (tEM), CCVs and other clathrin assemblies are identified as a distinctly periodic pattern of the cytoplasmic membrane coat arising from clathrin-triskelion oligomerization [15–17]. In live cell fluorescence microscopy of all cell types investigated so far, CME is characterized by the high dynamics that are observed for vesicle formation driven by clathrin triskelion oligomerization with an average life time of coats between 45-80s [18]. The dynamics of clathrin coats are further increased by constant exchange of individual triskelia throughout formation of the coated pit and the CCV to allow the reorganization from hexagons into pentagons as membrane curvature increases [14]. Active retrograde transport of PM-derived uncoated CCVs to endosomes adds a spatial dimension to the complex dynamics of CME.

In line with the endocytic nature of PV organelles, 3 protein complexes conserved in *Giardia* and associated with the endocytic machinery show a discrete localization in the cortical area of trophozoites by fluorescence microscopy: clathrin heavy chain (*Gl*CHC), subunits of the AP2 heterotetramer (*Gl*AP2) and *Giardia* dynamin related protein (*Gl*DRP) [3, 19]. These factors are key components of the clathrin-dependent endocytic machinery in higher eukaryotes and protozoa alike. However, no clathrin coated transport intermediates have been detected in *Giardia* by electron- or fluorescence microscopy. In terms of transport from the PM to PVs this is in line with the very short distances involved (~50 nm). Moreover, bulk fluid phase endocytosis into PVs has been demonstrated by time-lapse microscopy and appears to be independent of AP2 and dynamin [3, 19]. In addition, genes coding for regulatory factors involved in the dynamic formation and disassembly of coated pits and CCVs at the PM (i.e. clathrin light chain, HSC70, auxillin) cannot be identified, either because they are absent from the *Giardia* genome or because their sequence has diverged beyond recognition. Taken together, an important consequence of evolving a dedicated organelle (the ventral disk) for epithelial attachment may be remodeling of the endosomal organelle system and redistribution to the cortical region of trophozoites. In turn, given the proximity of PVs to the PM, these adaptations would have made dynamic CCV-mediated endocytic transport redundant and favored PV evolution towards fixed cortical compartments. This raises questions concerning the function of clathrin, its localization, and recruitment to membranes. To address these questions we performed a functional and structural characterization of conserved components of the giardial endocytic system. Extensive electron tomography revealed that the endocytic system of trophozoites is a network of elongated tubular compartments that may communicate with the extracellular environment by fusion at invaginations of the PM. On a molecular level, our data indicated that clathrin assemblies are small and focal clusters with no measurable turnover and exclusive cortical localization distal to PVs. To investigate the nature of these unusually static assemblies we generated an extended *Gl*CHC interactome which included conserved factors as well as new candidate adaptor proteins and a putative clathrin light chain (CLC). To our knowledge, this is the first report describing static clathrin heavy chain assemblies as a non-dynamic structural component of an endocytic system. Since formation of CCVs is conserved in all other eukaryotes, this suggests a novel function for clathrin and associated factors in the context of ecological specialization driven by host-pathogen interaction.

## Materials and Methods

### *Giardia* cell culture, induction of encystation and transfection

*G*. *lamblia* WBC6 (ATCC catalog number 50803) trophozoites were cultured and harvested applying standard protocols [20]. Induction of encystation was done by the two-step method as previously described [21, 22]. Transgenic parasites were generated using standard protocols for the electroporation of linearized pPacV-Integ-based plasmid vectors amplified *E*. *coli* as described in [23]. Transgenic cells were selected for puromycin resistance. Once selected, trophozoites were cultured and analyzed without antibiotics.

### Construction of expression vectors

Primer sequences of all oligonucleotides used for cloning are listed in S2 Table.

All genetically modified proteins in this manuscript were expressed under the control of the corresponding endogenous promoters except the 3 constructs for the truncation experiments of ORF4259 that were controlled by the inducible cyst wall protein 1 promoter.C-terminally hemagglutinin (HA)-tagged proteins were generated using a modified pPacV-Integ [24] with additional restrictions sites (Vector map in S2 Fig). All other cloning strategies were based on the previously described pPacV-Integ [24].

### Co-immunoprecipitation with limited cross-linking

*G*. *lamblia* WBC6 and transgenic trophozoites expressing N- or C-terminally HA tagged bait proteins were harvested and correct subcellular distribution of bait proteins was confirmed by immunofluorescence assay. After harvesting parasites were washed in 50 ml of cold phosphate buffer saline solution (PBS) and adjusted to 2x10^7^ cells ml^-1^ in PBS (VWR Prolabo). A titration assay was applied to determine the appropriate concentration (0.4mM) of the cell-permeable, lysine-reactive crosslinker Dithiobis[succinimidyl propionate] (DSP, also called Lamont’s Reagent). DSP is a particularly suitable crosslinking reagent to interrogate labile protein-protein interactions [25–28]. For the co-immunoprecipitation (co-IP) assays, 10^9^ parasites were resuspended in 20 ml 0.4mM DSP (in PBS) and incubated for 30 minutes at room temperature (RT) in the presents of 1 mM phenylmethylsulfonyl fluoride (PMSF; SIGMA, Cat. No. P7626). Thereafter cells were washed with PBS and quenched in 20 ml 100 mM glycine in PBS for 15 minutes at RT. Cells were pelleted and resuspended in 5ml of PBS supplemented with 2 mM PMSF and 1 x Protease Inhibitor Cocktail (PIC, Cat. No. 539131, Calbiochem USA) and sonicated twice using a Branson Sonifier with microtip (Branson Sonifier 250, Branson Ultrasonics Corporation) with the following settings: 60 pulses, 2 output control, 30% duty cycle, and 60 pulses, 4 output control, 40% duty cycle, respectively. The sonicate was centrifuged (14,000 *x g*, 10 minutes, 4°C) before the soluble protein fraction was diluted 1:1 with PBS supplemented with 2% Triton X -100 (TX-100) (Fluka Chemicals) and 60 μl anti-HA agarose bead slurry (Pierce, product # 26181). For the binding reaction of the HA-tagged proteins and the beads, samples were incubated for 2h on a rotating wheel at 4°C. Subsequently, beads were washed 4 times with 3 ml Tris-Buffered Saline (TBS) supplemented with 0.1% TX-100 and once with 3 ml PBS by pulse-centrifugation at 4°C. Loaded beads were transferred to a spin column (Pierce spin column screw cap, product # 69705, Thermo Scientific) in 350ul PBS, centrifuged for 10s at 4°C and eluted in 30ul PBS. To reverse the crosslinking, eluted fractions were supplemented with 100mM Dithiothreitol (DTT; 100mM; Thermo Scientific, Cat. # RO861) and incubated for 30min at 37°C before boiling for 5min and centrifugation (14,000 x g, 10 minutes, RT).

### Protein analysis and sample preparation for mass spectrometry-based protein identification

Analysis of input, flow-through, and eluted fractions was performed by SDS-PAGE on 10% polyacrylamide gels under reducing conditions, (molecular weight marker Cat. No. 26616, Thermo Scientific, Lithuania). Immunoblotting was done as previously described in [29]. Gels for mass spectrometry (MS) analysis were stained using Instant Blue (Expedeon, Prod. # ISB1L) and washed with ultrapure water.

### Mass spectrometry, protein identification and data storage

Stained gel lanes were cut into 8 equal sections. Each section was further diced into smaller pieces and washed twice with 100 μl of 100 mM ammonium bicarbonate/ 50% acetonitrile for 15 min at 50°C. The sections were dehydrated with 50 μl of acetonitrile. The gel pieces were rehydrated with 20 μl trypsin solution (5ng/μl in 10mM Tris-HCl/ 2mM CaCl_2_ at pH8.2) and 40μl buffer (10mM Tris-HCl/ 2mM CaCl_2_ at pH8.2). Microwave-assisted digestion was performed for 30 minutes at 60°C with the microwave power set to 5 W (CEM Discover, CEM corp., USA). Supernatants were collected in fresh tubes and the gel pieces were extracted with 150μl of 0.1% trifluoroacetic acid/ 50% acetonitrile. Supernatants were combined, dried, and the samples were dissolved in 20μl 0.1% formic acid before being transferred to the autosampler vials for liquid chromatography-tandem MS (injection volume 7 to 9 μl). Samples were measured on a Q-exactive mass spectrometer (Thermo Scientific) equipped with a nanoAcquity UPLC (Waters Corporation). Peptides were trapped on a Symmetry C18, 5μm, 180μm x 20mm column (Waters Corporation) and separated on a BEH300 C18, 1.7μm, 75μm x 150mm column (Waters Corporation) using a gradient formed between solvent A (0.1% formic acid in water) and solvent B (0.1% formic acid in acetonitrile). The gradient started at 1% solvent B and the concentration of solvent B was increased to 40% within 60 minutes. Following peptide data acquisition, database searches were performed using the MASCOT search program against the *G*. *lamblia* database (http://tinyurl.com/37z5zqp) with a concatenated decoy database supplemented with commonly observed contaminants and the Swissprot database to increase database size. The identified hits were then loaded onto the Scaffold Viewer version 4 (Proteome Software, Portland, US) and filtered based on high stringency parameters (minimal mascot score of 95% for peptide probability, a protein probability of 95%, and a minimum of 2 unique peptides per protein (95_2_95) or on slightly relaxed stringency parameters (95_2_50). Access to raw MS data is provided through the ProteomeXchange Consortium on the PRIDE platform [30]. Data are freely retrievable using project accession number PXD003718 and project DOI 10.6019/PXD003718. Accession numbers for datasets derived from bait-specific and corresponding control co-IP MS analyses are the following: **62506**: truncated HA-tagged ORF4259 as bait and non-transfected cells as control; **62507**: HA-tagged ORF4259 and non-transfected cells as control; **62508**: HA-tagged ORFs 17304 and 21423 and non-transfected cells as control; **62509**: HA-tagged ORF102108 and non-transfected cells as control; **62510**: native coIP on HA-tagged ORF102108; **62511**: HA-tagged ORF16595 and non-transfected cells as control; **62512**: HA-tagged ORFs 15411 and 16653; **62513**: HA-tagged ORFs 14373 and 7723.

### *In silico* co-immunoprecipitation dataset analysis

Analysis of primary structure and domain architecture of putative components of the giardial clathrin heavy chain protein network was performed using the following tools and databases: SMART (http://smart.embl-heidelberg.de/) for prediction of patterns and functional domains, pBLAST for protein homology detection (http://blast.ncbi.nlm.nih.gov/Blast.cgi?PAGE=Proteins), HHPred (http://toolkit.tuebingen.mpg.de/hhpred) for protein homology detection based on Hidden Markov Model (HMM-HMM) comparison, PSORTII (http://psort.hgc.jp/form2.html) for subcellular localization prediction, TMHMM (http://www.cbs.dtu.dk/services/TMHMM/) for transmembrane helix prediction, and the Giardia Genome Database (http://giardiadb.org/giardiadb/) to extract organism-specific information like protein expression levels, predicted molecular sizes and nucleotide/protein sequence. The *Gl*CHC co-IP dataset was filtered using a dedicated ctrl. co-IP dataset generated with non-transgenic WB parasites. Additional bait-specific co-IP datasets were compared to the average of four biological replicates of the ctrl.co-IP dataset.

### Immunofluorescence analysis (IFA) and microscopy

Sample preparation for immunofluorescence based wide field and laser scanning confocal microscopic (LSCM) analysis of transgenic cell lines was done as described previously in [23, 29]. Nuclear DNA was labeled with 4',6-diamidino-2-phenylindole (DAPI). Proteins were detected with either the anti-HA antibody (clone 3F10, monoclonal antibody from Roche) or with monospecific antibodies raised against *Gl*CHC. Cells (≥100/sample) were generally imaged at maximum width, where the nuclei and the bare-zone are at maximum diameter. Huygens Professional (Scientific Volume Imaging) was used to deconvolve image stacks of optical sections. Three-dimensional reconstructions, isosurface models and fluorescence lifetime analysis, and signal overlap quantification (Mander’s coefficient) in volume images of reconstructed stacks were performed using IMARIS x64 version 7.7.2 software suite (Bitplane AG).

### Live-cell microscopy and fluorescence recovery after photobleaching (FRAP)

For live-cell imaging trophozoites (≥40/sample) expressing *Gl*CHC::GFP, *Gl*17304::GFP, *Gl*4259::GFP or GFP::*Gl*DRP were harvested, washed once in cold PBS before re-suspending in PBS supplemented with 5mM glucose (Cat. No. 49139, Fluka) and 0.1mM ascorbic acid (Cat. No. 95209, Fluka) at pH 7.1. FRAP and time-lapse series were performed as described previously [3, 29].

### PV labelling using fluid-phase and membrane-associated markers

Fluid-phase uptake assays for *G*. *lamblia* and *S*. *vortens* were performed as described previously [3] using dextran 10,000Da coupled to either Texas Red (dextran-TxR) (Cat. No. D-1863, Thermo Fisher Scientific) or Oregon Green (dextran-OG) (Cat. No. D-7171, Thermo Fisher Scientific) fluorophores, both at 1mg/ml final concentration. Labelling of the plasma membrane was performed with cholera toxin b subunit coupled to Alexafluor 594 fluorophore (CTxb-594) (C22842, Thermo Fisher Scientific) at a final concentration of 10μg/ml [31].

### Super resolution (gSTED) microscopy

Sample preparation was done as described for wide field microscopy and LSCM. For imaging, samples were mounted in ProLong Gold antifade reagent (Cat. No. P36934, Thermo Fisher Scientific). Super resolution microscopy was performed on a LSCM SP8 gSTED 3X Leica (Leica Microsystems). Nuclear labeling was omitted due to possible interference with the STED laser. Further data processing and three dimensional reconstructions of image stacks were done as described for LSCM.

### DAB staining in APEX2 expressing cells

Transgenic trophozoites expressing *Gl*CHC-APEX2-2HA, *Gl*4259-APEX2-2HA or APEX2-2HA–*Gl*DRP were harvested and washed with PBS. Fixation was done in 2.5% EM grade glutaraldehyde in cacodylate buffer (100mM cacodylate (Cat. No. 20838, Fluka), 2mM CaCl2 (Cat. No. 21097, Fluka) in PBS) for 1h at RT. Samples were washed twice before and after quenching for 5min in 20mM glycine/cacodylate buffer. For staining, the cells were resuspended in 500ul substrate solution containing 1.4mM DAB tetrahydrochloride (Cat. No. D5637, Sigma) with 0.3mM H2O2 (Cat. No. H1009, Sigma) in cacodylate buffer and incubated between 1 and 15min. To stop the reaction, samples were washed trice in cacodylate buffer and prepared as described for TEM imaging. The pcDNA3 APEX2-NES vector was a gift from Alice Ting (Addgene plasmid # 49386).

### Preparation of chemically fixed, DAB stained cells

DAB stained cell suspensions were post-fixed with 1% aqueous OsO4 for 1 hour on ice, subsequently rinsed three times with pure water and dehydrated in a sequence of ethanol solutions (70% up to 100%), followed by incubation in 100% propylene oxide and embedding in Epon/Araldite (Sigma-Aldrich, Buchs, Switzerland). Samples were polymerized at 60°C for 24h. Thin sections were imaged without post-staining as well as after post-staining with aqueous uranyl acetate (2%) and Reynolds lead citrate.

### Preparation of native cell suspensions by high-pressure freezing

For subsequent TEM and FIB-SEM analysis, 30μl of concentrated *Giardia lamblia* suspension was pipetted onto a carbon coated 6mm Sapphire discs (100μm thickness) set up in a special middle plate for high-pressure freezing of adherent cell cultures in an EM HPM 100 high-pressure freezing system (Leica Microsystems, Vienna, Austria). After an incubation time of 5 minutes letting the cells attach to the surface, the suspension was drawn off with a filter paper and the cells were covered with a 6mm aluminum specimen carrier wetted with 1-hexadecene and the 100μm cavity facing the cells. Finally, a 200μm-thick spacer ring (diameter 6mm) was added on top and the specimen immediately high-pressure frozen without using alcohol as synchronization fluid. Freeze-substitution was carried out in water-free acetone with 1% OsO4 for 8h at -90°C, 7h at -60°C, 5h at -30°C, 1h at 0°C, with transition gradients of 30°C per hour. Samples were rinsed twice with acetone water-free, stained with 1% uranyl acetate in acetone (stock solution: 20% in MeOH) for 1h at 4°C, rinsed twice with water-free acetone and embedded in Epon/Araldite: 66% in acetone overnight, 100% for 1h at RT and polymerized at 60°C for 20h. Thin sections were post-stained with Reynolds lead citrate.

All thin sections were imaged in a CM 100 transmission electron microscope (FEI, Eindhoven, The Netherlands) at an acceleration voltage of 80kV using a Gatan Orius 1000 CCD camera (Gatan, Munich, Germany).

For subsequent FIB-SEM tomography, an Epon/Araldite block containing *Giardia* cells was mounted on a regular SEM stub using conductive carbon and coated with 10 nm of carbon by electron beam evaporation to render the sample conductive. Ion milling and image acquisition was performed simultaneously in an Auriga 40 Crossbeam system (Zeiss, Oberkochen, Germany) using the FIBICS Nanopatterning engine (Fibics Inc., Ottawa, Canada). A large trench was milled at a current of 20nA and 30kV, followed by fine milling at 600pA and 30kV during image acquisition with an advance of 5nm per image. Prior to starting the fine milling and imaging, a protective platinum layer of approximately 300nm was applied on top of the surface of the area of interest using the single gas injection system at the FIB-SEM. SEM images were acquired at 1.7kV (30μm aperture) using an in-lens energy selective backscattered electron detector (ESB) with a grid voltage of 1.4kV, and a dwell time of 40μs. The pixel size was set to 5nm and tilt-corrected to obtain isotropic voxels.

The final image stack was cropped to the area of interest using the ImageJ image processing package Alignment of the image stack was performed with the Sift plugin. For 3D reconstructions, organelle membranes and plasma membrane invaginations were traced manually in the interactive learning and segmentation toolkit (Ilastik) [32] and subsequently rendered in Imaris (Bitplane AG, Zurich, Switzerland).

For subsequent Cryo-SEM, a concentrated *Giardia* cell suspension was sandwiched between two interlocking gold specimen carriers (Leica Microsystems) and immediately high-pressure frozen using a EM HPM100 without alcohol as synchronization fluid. After freezing, the sandwich was mounted under liquid nitrogen on a designated specimen holder for freeze fracturing in the VCT 100 cryopreparation box and transferred onto the cold stage of a BAF 060 freeze-fracturing device using the VCT 100 cryotransfer system (Leica Microsystems). The specimen was fractured at -120°C by removing the top carrier with the hard metal knife supplied with the BAF 060 device. The specimen was heated to -105°C for 5 minutes for sublimation and coated with 2.5nm platinum/carbon by electron beam evaporation at an angle of 45° unidirectionally and with 2.5nm platinum/carbon at 45° using stage rotation (40rpm). The specimen was retracted into the transfer shuttle of the VCT 100 system and transferred under high vacuum onto the cryostage in the SEM (Auriga 40 Crossbeam system, Zeiss). Specimens were imaged at 115°C (the saturation water vapor pressure of the specimen corresponding to the vacuum in the chamber of 5x10-^7^mbar) and at an acceleration voltage of 5kV using the in-lens secondary electron detector.

### Structural analysis using iTASSER

*Ab initio* prediction of hypothetical 3D models for CLC homologues and *Gl*4259 was done using i-TASSER (http://zhanglab.ccmb.med.umich.edu/I-TASSER/) [33–35]. Ten annotated CLC protein sequences were selected from several eukaryotic supergroups and each one subjected to pairwise alignment analysis with human clathrin light chain A isoform a. Modelled sequences were obtained by trimming at the variable N-terminus until the signature CLC motif was reached [36]. This resulted in the following truncated sequences: *M*. *musculus* (NP_001073853.1; residues 27–218), *S*. *cerevisiae* (EDN61754.1; residues 35–233), *D*. *melanogaster* (NP_524178.2; residues 28–219), *T*. *gondii* (KFG44257.1; residues 109–323), *T*. *resei* (XP_006967655.1; residues 27–236), *C*. *reinhardtii* (XP_001697531.1; residues 40–228), *C*. *elegans* (NP_504999.1, residues 36–226), *H*. *sapiens* (NP_001824.1; residues 27–218), *T*. *cruzi* (XP_819466.1; residues 31–215), *G*. *lamblia* (XP_001707073.1; residues 47–283). The final structures were displayed using VMD. Superimpositions of solved structures were done using a VMD MultiSeq plugin [37] and structural similarities were expressed as *Q*_*H*_ values.

## Results

### PVs form a network of elongated organelles with morphologically distinct PV-PM interfaces

**Fig 1:**
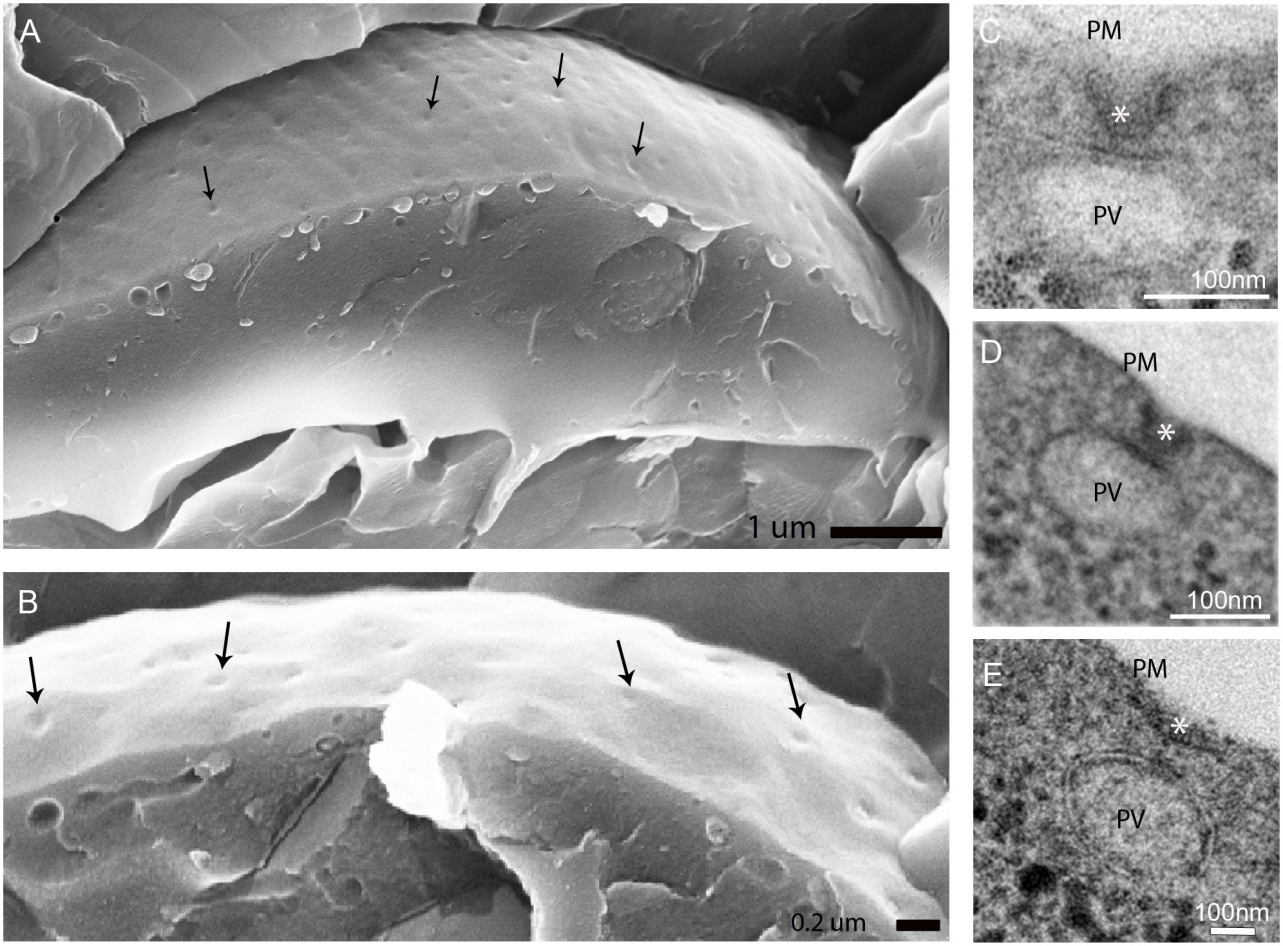
Scanning and transmission electron microscopy show PM-invaginations. (A, B) SEM micrographs show dimples on the parasite surface (arrows). (C-E) tEM micrograph highlights of peripheral vacuole-associated plasma membrane-invaginations. PV: PV lumen; PM: plasma membrane; asterisks: invaginations.

**Fig 2:**
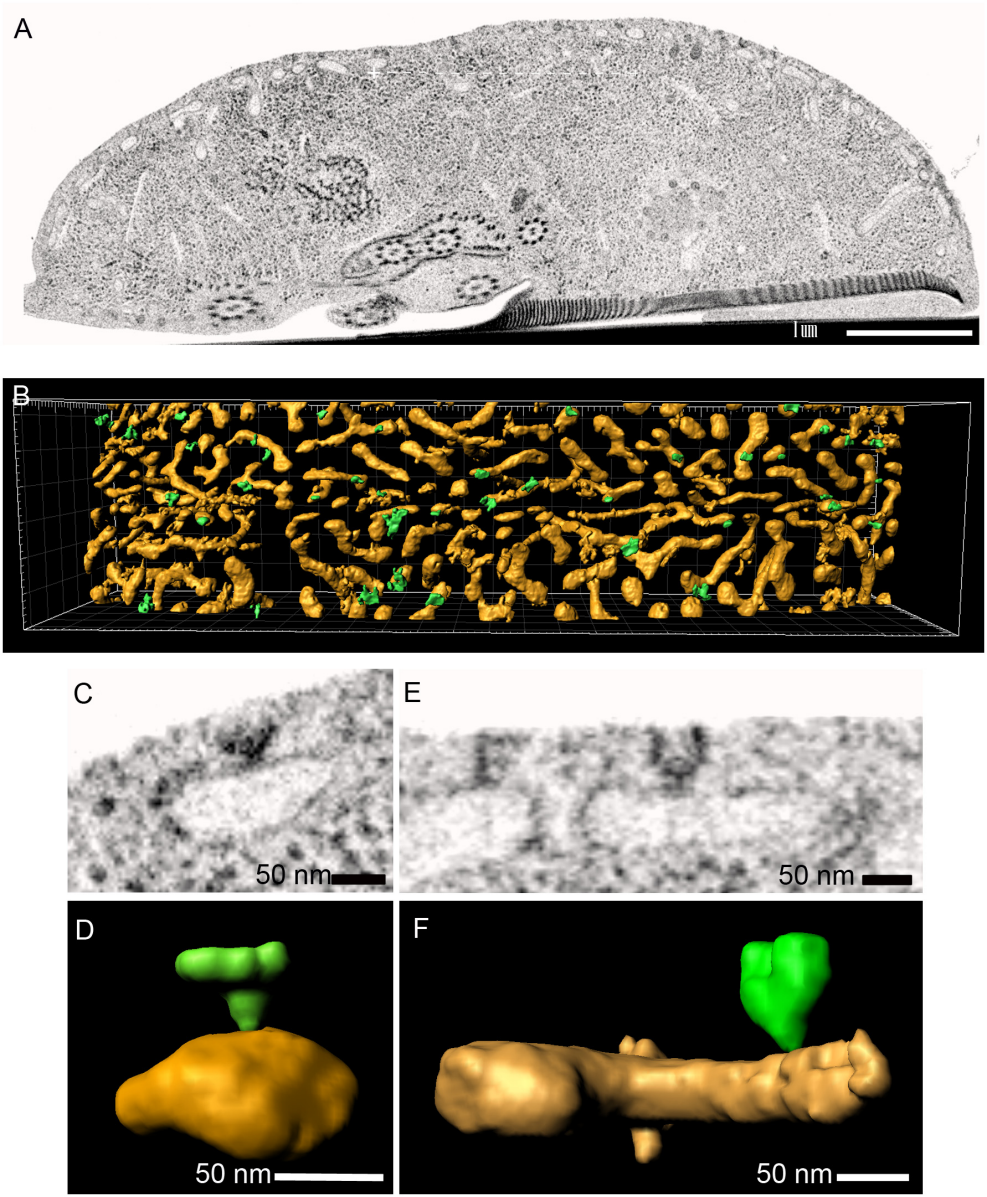
FIB-SEM based 3D rendering of PVs and PM-invaginations. (A) Representative image of the FIB-SEM stack used for rendering in (B,D,F). (B) Dorsal view of the PV-network (yellow) with PM-associated invaginations (green). (D, F) Reconstruction of individual organelles with PM-associated invaginations as detected in FIB-SEM (C, E).

Despite extensive EM studies of the giardial endocytic system, a detailed morphological characterization of PVs and associated structures was not yet done. We showed previously that a fluid phase maker, fluorescently labeled dextran-TxR, is taken up in bulk by PVs and expelled again in a cycle of approximately 20 minutes [3, 38]. This reflects rapid and unselective uptake of large extracellular fluid volumes across the PM into PVs, calling for either direct *i*.*e*. membrane-to-membrane, or indirect *i*.*e*. vesicle-mediated fusion events involving the plasma and PV membranes. To characterize the morphology of PVs and structures at the PV—PM interface that might mediate continuity between these compartments, we applied tEM, SEM and FIB-SEM microscopy on chemically fixed or high-pressure-frozen (HPF) cells. The intact surface of freeze-fractured trophozoites reveals evenly distributed small surface indentations on the extracellular side of the PM (Fig 1A and 1B). On the cytoplasmic side, PVs are clearly visible as oval or elongated tubular compartments just underneath the PM. To determine whether these surface indentations might correspond to PM-PV membrane contact sites we used TEM imaging. Thin lateral sections (70nm) revealed PM invaginations of up to ~50-70nm which were always associated with PVs, in line with earlier observations [4] (Fig 1C–1E). To define the architecture of the cortical PV organelle system in relation to the observed PM invaginations more precisely in three dimensions, we used FIB-SEM tomography of HPF-fixed trophozoites (Fig 2A). Surface-rendered reconstructions revealed a distinctly tubular nature of the cortical organelle array which had not been appreciated before. We also noted that some organelles reached deep in the cytoplasm (Fig 2A). Membrane tracing and subsequent surface rendering also showed that funnel-shaped PM invaginations always associated with one PV organelle, averaging one invagination/PV (Fig 2B–2F).

To address the question whether fusion events occur between the PM and PV membrane we first saturated PVs with dextran-Oregon Green and then labeled the PM with Alexafluor 594-conjugated cholera toxin-B subunit (CTxb-594) to follow the fate of PM lipid components over time [31]. Within 40 min, the vast majority of CTxb-594 label re-located to fluorescently-labeled PVs (S1A and S1C Fig). Translocation of CTxb-594 signal from the PM to PVs is consistent with the steady increase over time in Mander’s coefficient of signal overlap (S1B Fig). This is direct evidence for lipid exchange between PM—PV membranes most likely at specific contact sites, with similar dynamics to previously measured turnover of PV fluid phase content [3].

Taken together, this suggests a direct communication of the elongated endocytic organelles with the extracellular environment by way of PM-invaginations forming continuities with PV membranes. This raised questions about the molecular underpinnings of these membrane deformations and the nature of the electron-dense material at the PM-PV interfaces (Fig 1C–1E).

### *Giardia lamblia* clathrin heavy chain is organized in regularly distributed, focal assemblies at the PV-PM interface

**Fig 3:**
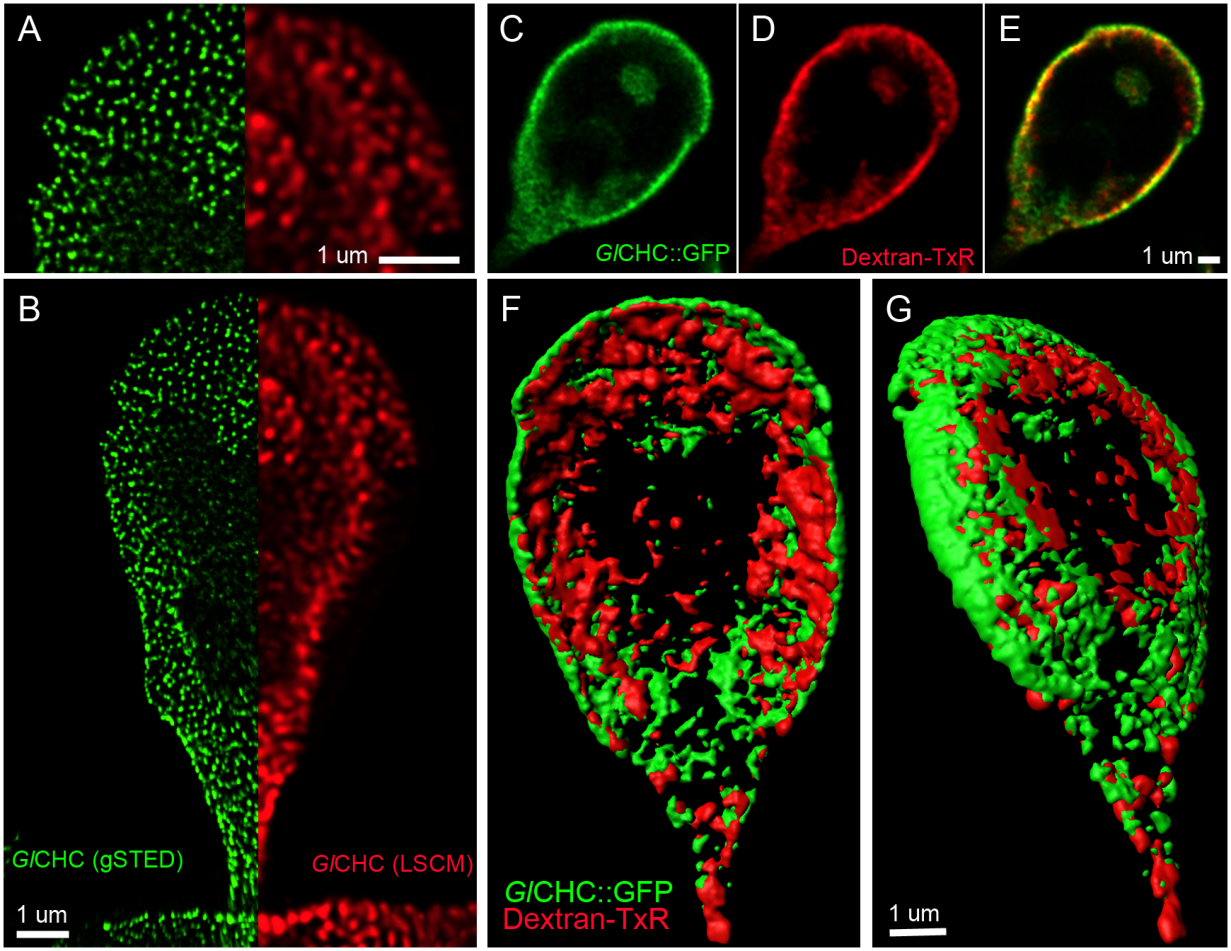
GlCHC reporters as seen by gSTED microscopy, standard LSCM and co-labeled with dextran-TxR. (A, B) The improved resolution of gSTED super resolution microscopy (green signal) compared to a standard LSCM signal (red signal) of the HA-tagged *Gl*CHC reporter in fixed cells indicates that *Gl*CHC assembly size is in the range of 50nm (A: inset of B). (C-E) Single optical sections of GFP-tagged *Gl*CHC-reporters (C) co-labeled with the fluid phase marker dextran-TxR (D) (E: merged images). (F, G) Surface rendering of optical sections from the cell shown in C-E shows that the *Gl*CHC-GFP reporter (green) localizes distal to the PV-marker dextran-TxR (F: ventral view, G: dorso-lateral view).

**Fig 4:**
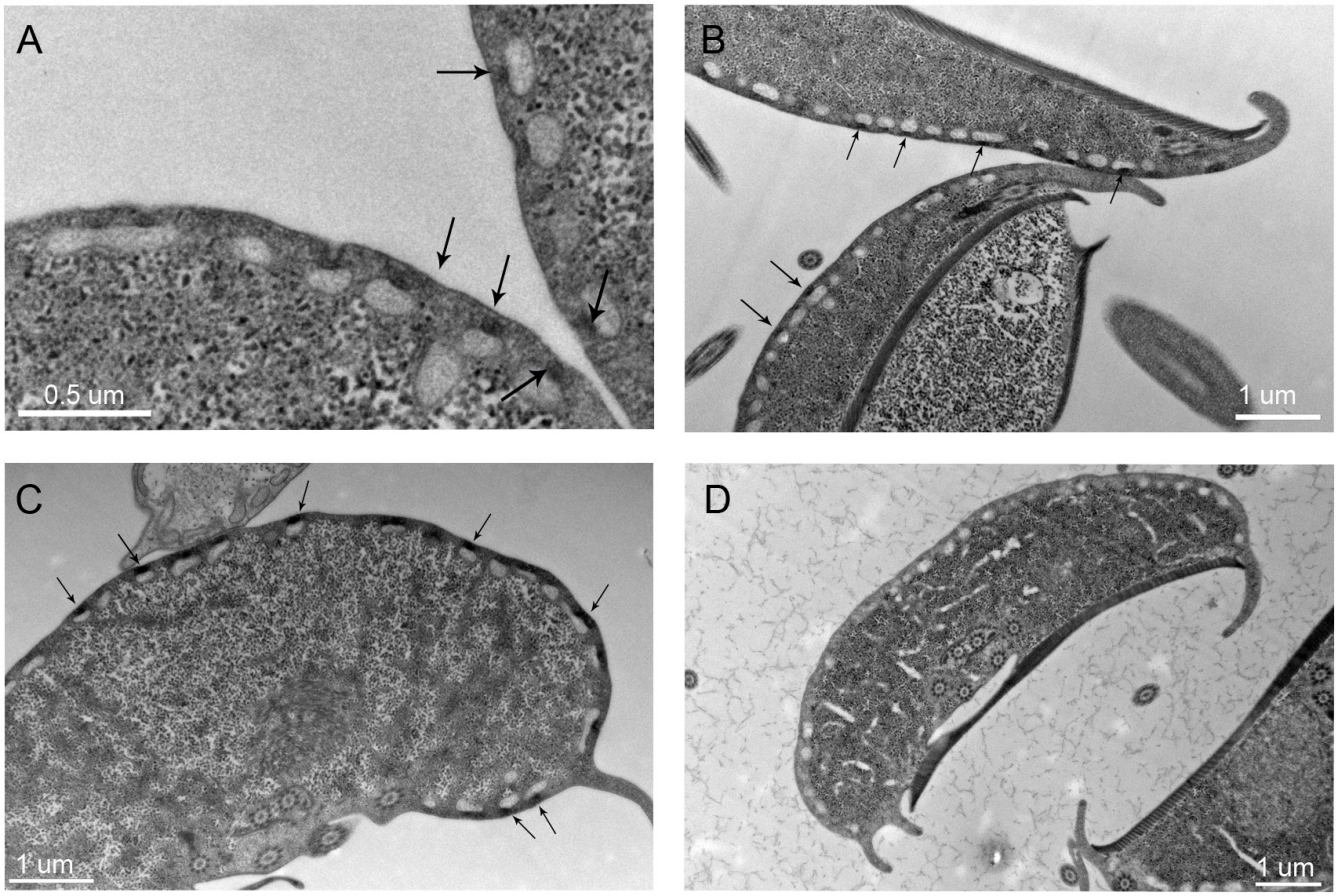
Localization of the GlCHC APEX2-2HA reporter. TEM images of *Gl*CHC-APEX2-2HA expressing cells after exposure to DAB for 1min (A), 5min (B) and 15min (C). In all conditions the signal specifically localizes to the PPI. Arrows indicate reporter-specific signals. (D) Non-transfected control cell exposed to DAB for 15min.

CCVs forming from coated pits at the PM are not detected in tEM images of *Giardia* trophozoites. Both the giardial clathrin heavy chain (*Gl*CHC) and the dynamin-related protein (*Gl*DRP) localize with significant signal overlap at punctate structures in the trophozoite cell cortex containing PVs by confocal fluorescence microscopy [3]. We investigated the nature of these small *Gl*CHC assemblies more precisely using gated STED super resolution confocal microscopy. *Gl*CHC-HA was detected in uniformly sized, small (<50nm) assemblies, which were regularly distributed in the cortical area of the cell (Fig 3A and 3B). In line with the absence of CCVs in tEM, the small size and distribution of the CHC assemblies visualized by STED microscopy was not consistent with coated vesicles (average size 80-100nm [11, 39–41]). To analyze the *Gl*CHC distribution in relation to PV-organelles we imaged endocytosed dextran-TxR with *Gl*CHC::GFP in living cells using confocal microscopy. Single optical sections and three dimensional reconstructions of image stacks consistently showed localization of the *Gl*CHC::GFP signal distal to the endocytosed dextran-TxR at the PM-PV interface (PPI) (Fig 3C–3G). The localization of clathrin assemblies on a single organelle level was further specified by an ectopically expressed CHC construct tagged with APEX2, a genetically encoded enzymatic reporter for tEM [42, 43]. The *Gl*CHC::APEX2-2HA-specific signal obtained in tEM from three different labeling conditions consistently showed specific and focal localization of electron dense deposits at the PPI with each signal specifically correlated to a PV-organelle and increasing over time (Fig 4A–4C). Importantly, the experiment with the lowest exposure time (Fig 4a) emphasized basket-like structures at the PPI, which completely bridged the ~70nm gap between the PM and the PV membranes and contacted both. Untransfected control cells exposed to the substrate for the maximal incubation time of 15 min (Fig 4D) showed no APEX-specific labelling, only the previously observed increased electron-density at invaginated regions where the PM is in close approximation to the PV membrane (compare also with Fig 1C and 1D and S3 Fig). The subcellular distribution of the APEX2-tagged reporter in immunofluorescence assays using the anti-HA antibody was completely consistent with incorporation in clathrin assemblies (S4A–S4D Fig). Taken together, the data provides evidence for the organization of CHC as distinctive assemblies that are associated with membrane invaginations akin to coated pits, but not transport vesicles. This fits with the absence of CCVs in the cell cortex by tEM, and suggests that CCVs do not occur in the giardial endocytic pathway.

### A *Gl*CHC reporter has no measurable turnover in focal assemblies

The absence of CCV formation at the PM suggested that *Gl*CHC assmblies might function to stabilize specialized regions of the PPI. Canonical CCVs have a lifetime of 45-80s (from emergence to fusion; [18]) and are highly dynamic, presenting constant exchange of CHC triskelia throughout all stages of CME [14]. Thus, rather than forming membrane vesicles for endocytic transport, giardial CHC assemblies might be more stable structures involved in membrane fusion events which physically connect the PV lumen to the extracellular environment, e.g. during fluid phase uptake. To address this, we quantified turnover of *Gl*CHC in assemblies at the PPI and measured the lifetime of assemblies in living cells.

**Fig 5:**
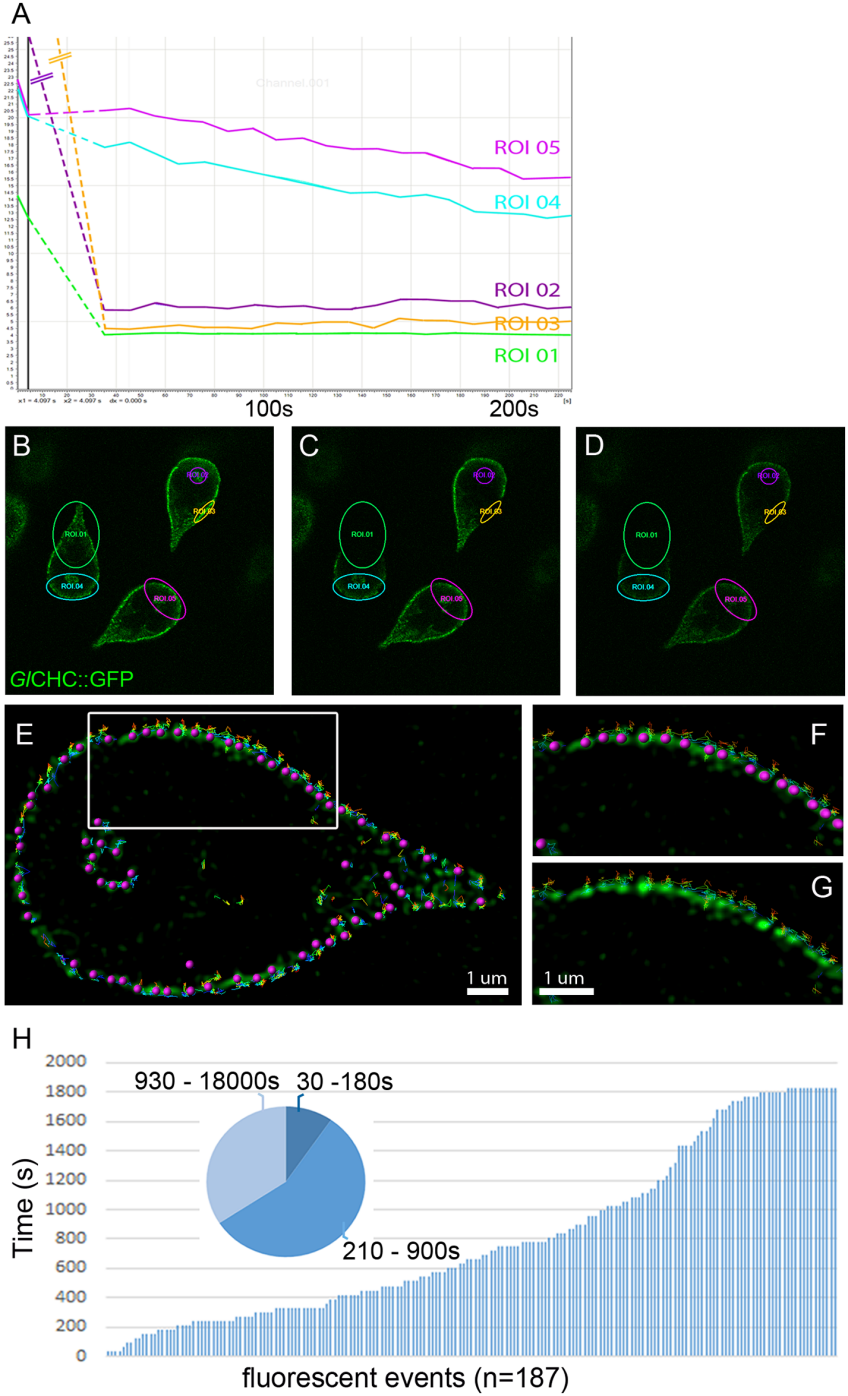
Dynamic and lifetime analysis of the GlCHC:GFP reporter. (A-D) FRAP and iFRAP analyses were done in the same field of view including an unbleached control cell (ROI05). FRAP analysis (ROI02, ROI03) shows no recovery whilst iFRAP analysis (ROI04) shows no loss of fluorescence after 225s, indicating no measurable turnover for the *Gl*CHC-GFP reporter. Pre-bleach (B), post-bleach t0 (C), post-bleach t220 (D). (E-H) Lifetime analysis of individual *Gl*CHC assemblies over 30min. The insets F and G show precise recognition of individual fluorescent events by the spot tracking tool in IMARIS. Clathrin assemblies are labelled in green whereas individual tracking spots are labelled in purple. (H) Analysis of 187 individual fluorescent events revealed lifetimes between 30-180s (9.65%), 210-900s (56.15) and 930-1800s (34.20%).

We used FRAP and inverse FRAP (iFRAP) to quantify turnover of a constitutively expressed *Gl*CHC::GFP reporter which localized exclusively to clathrin assemblies in trophozoites. Selectively photo-bleached assemblies decorated with GFP in regions of interest (ROIs) did not recover any fluorescence after 220 seconds (Fig 5A–5D; ROIs 01–03). Further observations for > 10 min did not reveal any increase of the signal in the bleached area that would indicate turnover of the reporter (S4E Fig). In line with this, iFRAP analysis of cells expressing *Gl*CHC::GFP demonstrated equal fluorescence loss over time as an unbleached control cell (Fig 5A–5D; ROIs 04 and 05).

Next, we analyzed the lifetime of individual *Gl*CHC-assemblies in cells expressing *Gl*CHC::GFP reporters by time-lapse microscopy. Resonance-scan live-cell microscopy (single focal plane imaging) was performed as 30min (60 frames) time lapse series. Statistical analysis of the spatio-temporal distribution of the *Gl*CHC::GFP signal (IMARIS spot tracking) showed that the labeled assemblies were spatially highly restricted, and that >90% had a lifetime that widely exceeded the maximal lifetime of canonical CCVs (~3min) (Fig 5E–5H). Of note, 34% of the GFP-labeled assemblies had a lifetime between 15 and ≥30 minutes which suggested a very high stability of the associated structures.

We asked if reduction of *Gl*CHC levels or interference with the composition of focal assemblies would alter their number or distribution. Although a single gene knockout (1 of 4 alleles) has been achieved as a proof of concept [44], *Giardia* is not amenable to complete knockouts and conditional complementation of essential genes. In addition, a *Gl*CHC knockdown using morpholinos [45] led to insufficient reduction of *Gl*CHC expression. As an alternative, we attempted to elicit a dominant-negative effect using a hub fragment (*Gl*CHC-hub, E1229–H1871). Strong conditional over-expression of the CHC hub fragment in HeLa cells inhibits formation of coated pits and CCVs [46]. Conditional over-expression of *Gl*CHC-hub resulted in correct localization of the HA-tagged reporter to focal assemblies. However, using a specific antibody to *Gl*CHC, we could not detect any obvious changes in the number and distribution of *Gl*CHC assemblies in transgenic trophozoites (S4F Fig).

Taken together, the FRAP data showed no measurable turnover of the *Gl*CHC::GFP reporter in assemblies whilst fluorescence lifetime analysis demonstrated their highly static nature. This result is in line with the absence of CCVs and with a missing uncoating motif at the C-terminal end of the protein (S5 Fig). The evidence presented so far supports a non-canonical role for *Gl*CHC in *G*. *lamblia*. The lack of measurable turnover in assemblies suggests a non-conventional system for recruitment of *Gl*CHC to membranes independent of endocytic stimuli.

### Native co-IP detects a putative, highly diverged clathrin light chain

The unusual static nature of the small focal *Gl*CHC assemblies without a role in the formation of transport vesicles at the PM strongly suggest significant differences in their composition as well as in recruitment of CHC to the membrane, compared to canonical CME complexes. CCV cages or any other regular clathrin lattice structures were not observed despite extensive imaging and state-of-the-art sample preparation methods for tEM, SEM, and FIB-SEM. Consistent with the lack of turnover in assemblies (Fig 5A–5D), the *Gl*CHC C-terminus does not present the highly conserved QLMLT motif for HSC70-mediated coat disassembly (S5 Fig) [47, 48]. The absence of a giardial HSC70 homolog and its cofactor auxillin in the genome underscore the scarcely dynamic nature of *Gl*CHC assemblies. However, genes coding for the heterotetrameric *Gl*AP2 complex and *Gl*DRP were identified in GiardiaDB (http://tinyurl.com/37z5zqp). Even taking into account the high degree of sequence divergence in *Giardia*, this is in marked contrast to other eukaryotes where 30–50 conserved proteins are involved in the formation of clathrin coats during CME [10, 11].

**Fig 6:**
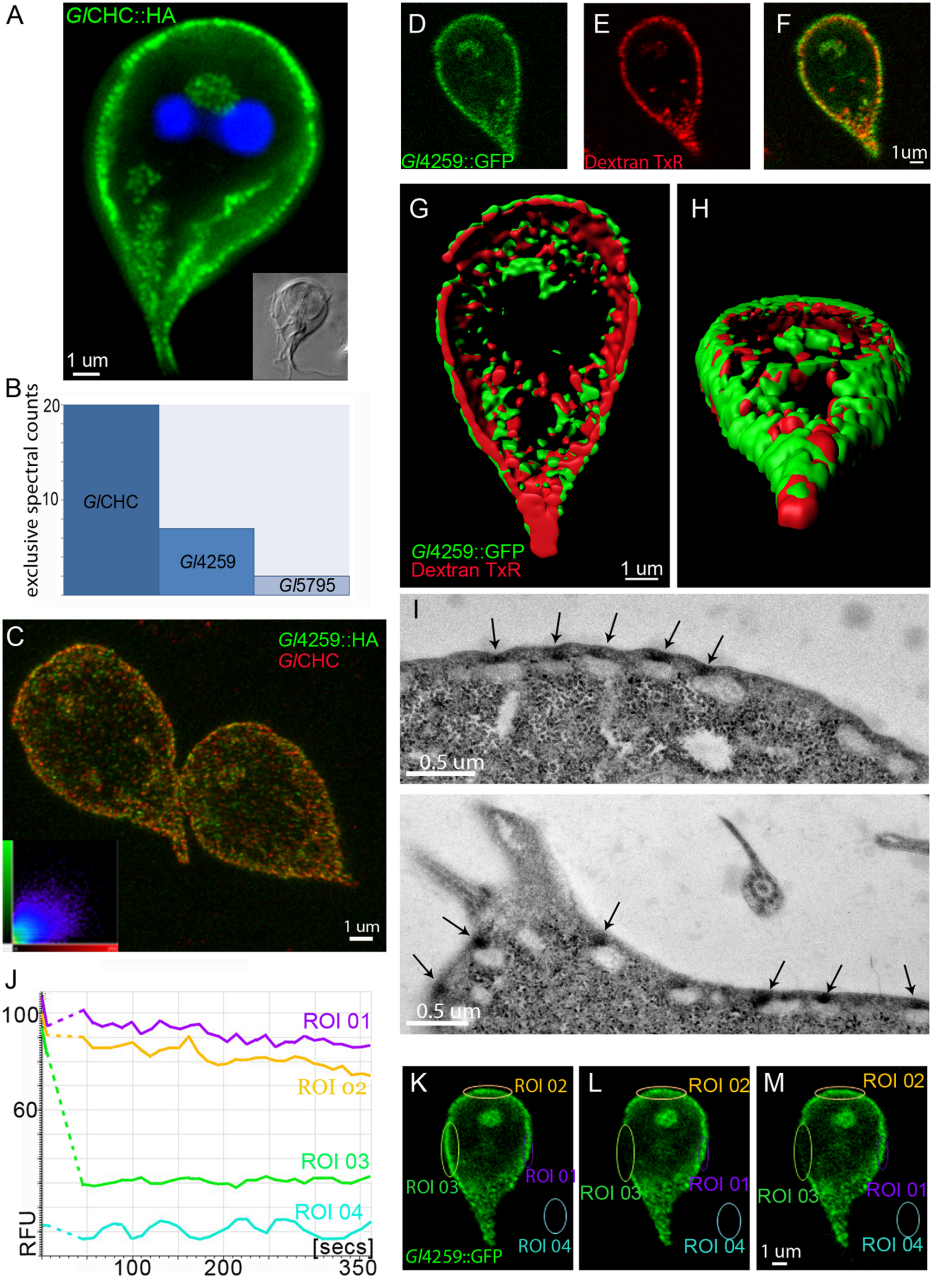
Proteomics and imaging-based analyses identify a putative clathrin light chain. (A) Subcellular localization of the HA-epitope tagged *Gl*CHC reporter in the cell cortex. (B) Exclusive spectral counts for all proteins detected at high stringency parameters in a native co-IP using the *Gl*CHC-HA bait. (C) Co-labeling of HA-tagged *Gl*4259 and endogenous *Gl*CHC shows considerable signal overlap in three dimensional reconstructions of image stacks (inset: co-localization scatter plot). (D-F) Single optical sections of GFP-tagged *Gl*4259-reporters (D) co-labeled with the fluid phase marker dextran-TxR (E) (F: merge image). (G, H) Surface rendering of optical sections from the cell shown in D-F, shows a more peripheral localization of *Gl*4259-GFP (green) compared to the signal for the PV-marker dextran-TxR (red) (G: ventral view, H: caudo-dorsal view). (I) Localization of the *Gl*4259-APEX2-2HA reporter to the PPI in TEM micrographs after 5min exposure to DAB. (J-M) FRAP analysis shows no recovery in the bleached ROI03 over time indicating no measurable turnover for the *Gl*4259-GFP reporter. Pre-bleach (K), post-bleach t0 (L), post-bleach t350 (M). RFU: relative fluorescence units.

To dissect the composition of clathrin assemblies in trophozoites we implemented a co-IP strategy in two stages using a constitutively expressed *Gl*CHC-HA bait protein. Exclusive localization of *Gl*CHC-HA at assemblies was confirmed by IFA in transgenic trophozoites (Fig 6A and S6 Fig). Subcellular distribution of the reporter matched the distribution of endogenous *Gl*CHC as shown previously by us and others [3, 19, 38]. We generated LC-MS/MS datasets from co-IP experiments of a transgenic cell line expressing the *Gl*CHC-HA bait (*Gl*CHC co-IP) and an untransfected line (ctrl. co-IP) as a control for unspecific binding and physical trapping.

First, a native co-IP experiment was performed to identify the strongest *Gl*CHC interactors (Fig 6B). We identified *Gl*4259, a protein of unknown function presenting no significant predictions for known functional domains, as the most prominent hit by far, and *Gl*5795, a protein containing a predicted leucine-rich domain. *Gl*4259’s strong interaction with *Gl*CHC was reflected in matching subcellular distributions (Fig 6C). Based on these data we hypothesized that *Gl*4259 was a highly diverged clathrin light chain (CLC). To investigate this further, we determined the subcellular localization of an epitope-tagged variant with respect to PVs. Fluorescence and electron microscopy analysis confirmed PPI-localization for HA-and APEX-tagged *Gl*4259 variants, respectively (Fig 6D–6H and 6I). The combined data strongly suggested that *Gl*4259 is an integral part of *Gl*CHC assemblies. Furthermore, similar to *Gl*CHC::GFP, a *Gl*4259::GFP reporter lacks turnover in FRAP experiments (Fig 6J–6M).

**Fig 7:**
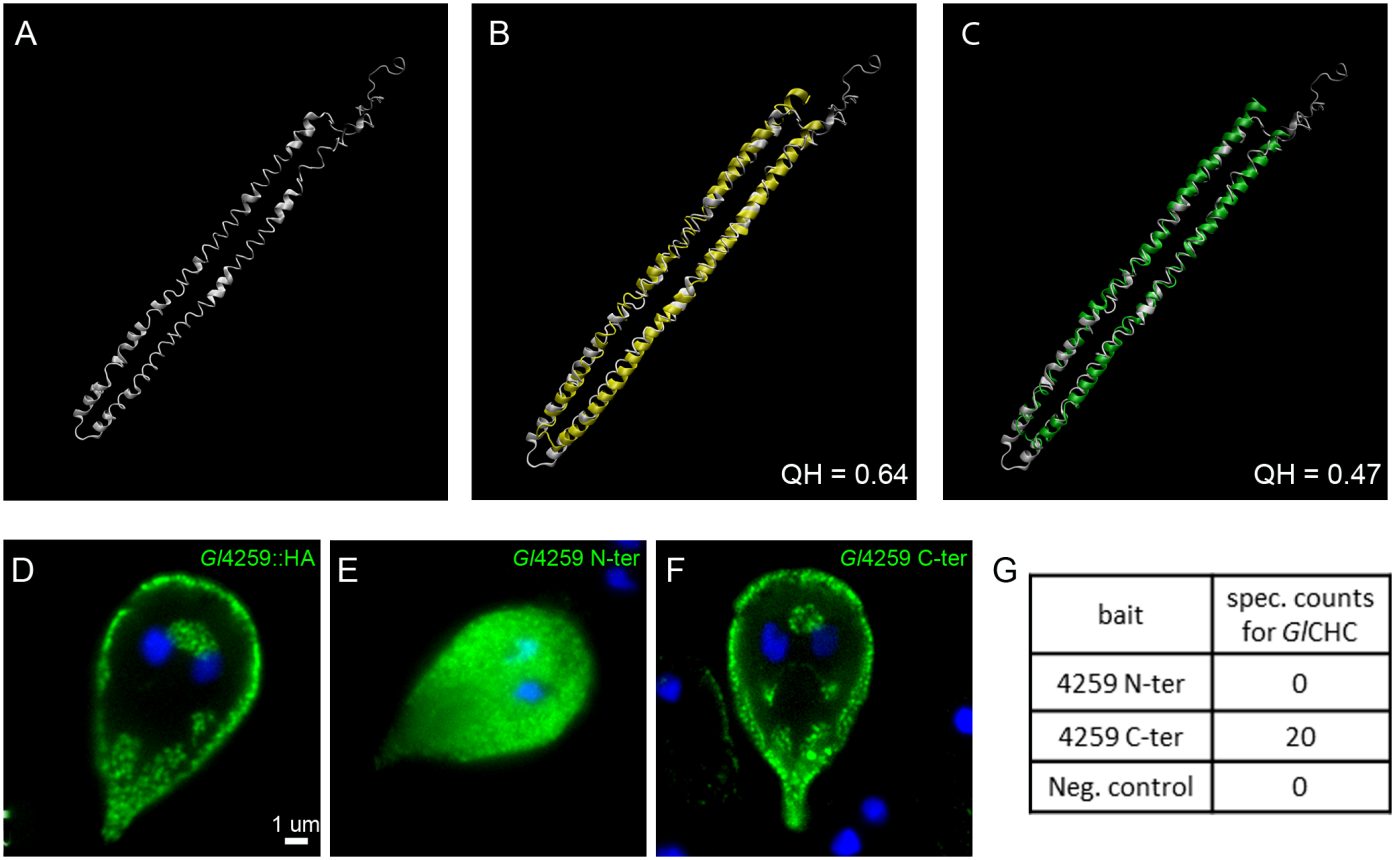
Functional and structural analysis of the putative clathrin light chain. (A-C) iTASSER *de novo* structural predictions for *Gl*4259 (A) showing a 64% and 47% structural overlap with iTASSER predictions for *H*. *sapiens* (B) and *T*. *brucei* (C) annotated clathrin light chains, respectively. (D-F) Representative images for the subcellular distribution of epitope-tagged truncated versions of *Gl*4259. The full length HA-tagged construct for *Gl*4259 (D) distributes at the cell cortex, in contrast the N-terminally truncated variant (E) is mislocalized to the cytosol, whilst the C-terminally HA-tagged truncated variant (F) localizes similar to A. (G) Co-IP experiments using the truncated variants shown in (B) and (C) as baits reveal interaction with *Gl*CHC only for the C-terminally truncated variant.

Because a solved structure of annotated full length CLC was not available, we used i-TASSER for protein structure prediction of *Gl*4259. To validate i-TASSER predictions for *Gl*4259, 9 additional annotated CLCs sequences were analyzed and compared with the models for *Gl*4259. i-TASSER provided 5 best-fit models for each sequence. For all modelled sequences, one out of five models showed protein folding into an elongated rod like structure (Fig 7A and S7 Fig). This is in line with published structural predictions for annotated CLC [49, 50]. Additionally, we measured a considerable structural overlap (46.5%–66.7.2%) for *Gl*4259 with the corresponding models of annotated CLC sequences (Fig 7B and 7C and S7 Fig). Functional analysis to determine if *Gl*4259 possessed CLC-like properties was based on several reports identifying either the C-terminus or a α-helical structure of the CLC middle domain to be responsible for CHC binding [50–53]. To test this we generated truncated versions of *Gl*4259 based on secondary structure analysis. A C-terminally truncated variant (L149 to K283) contained all predicted α-helices, while the N-terminally truncated variant (M1 to H148) contained none. Only the C-terminal fragment and the full-length control construct localized to CHC assemblies, whilst the N-terminal fragment was distributed throughout the cytoplasm (Fig 7D–7F). Additional analysis demonstrated protein-protein interactions between *Gl*CHC and the C-terminal fragment only (Fig 7G). Taken together, our results demonstrate that *Gl*4259 is a very strong *Gl*CHC-interacting protein with matching localization and turnover dynamics. Together with structural and functional data this strongly supports the hypothesis that *Gl*4259 represents a highly diverged giardial CLC.

### Co-IP assays identify conserved and novel *Gl*CHC-associated proteins

*Gl*CHC and *Gl*4295 form highly stable interactions at the PPI and are likely the main structural components of clathrin focal assemblies. However, CME pathways in other eukaryotes are based on a network of weak protein-protein interactions which regulate the tightly-controlled assembly and disassembly of clathrin coat components [54]. To test whether additional proteins could be identified as components of *Gl*CHC assemblies, we used the *Gl*CHC-HA variant as bait in a co-IP protocol adapted to stabilize weak protein-protein interactions by including a chemical cross-linking step prior to cell disruption and bait pull-down [55–57]. For this study we titrated the reversible, cell-permeable, lysine-reactive crosslinker Dithiobis[succinimidyl propionate] (DSP, also known as Lamont’s Reagent) to define optimal concentrations for limited cross-linking (S8 Fig) [25–28].

**Fig 8:**
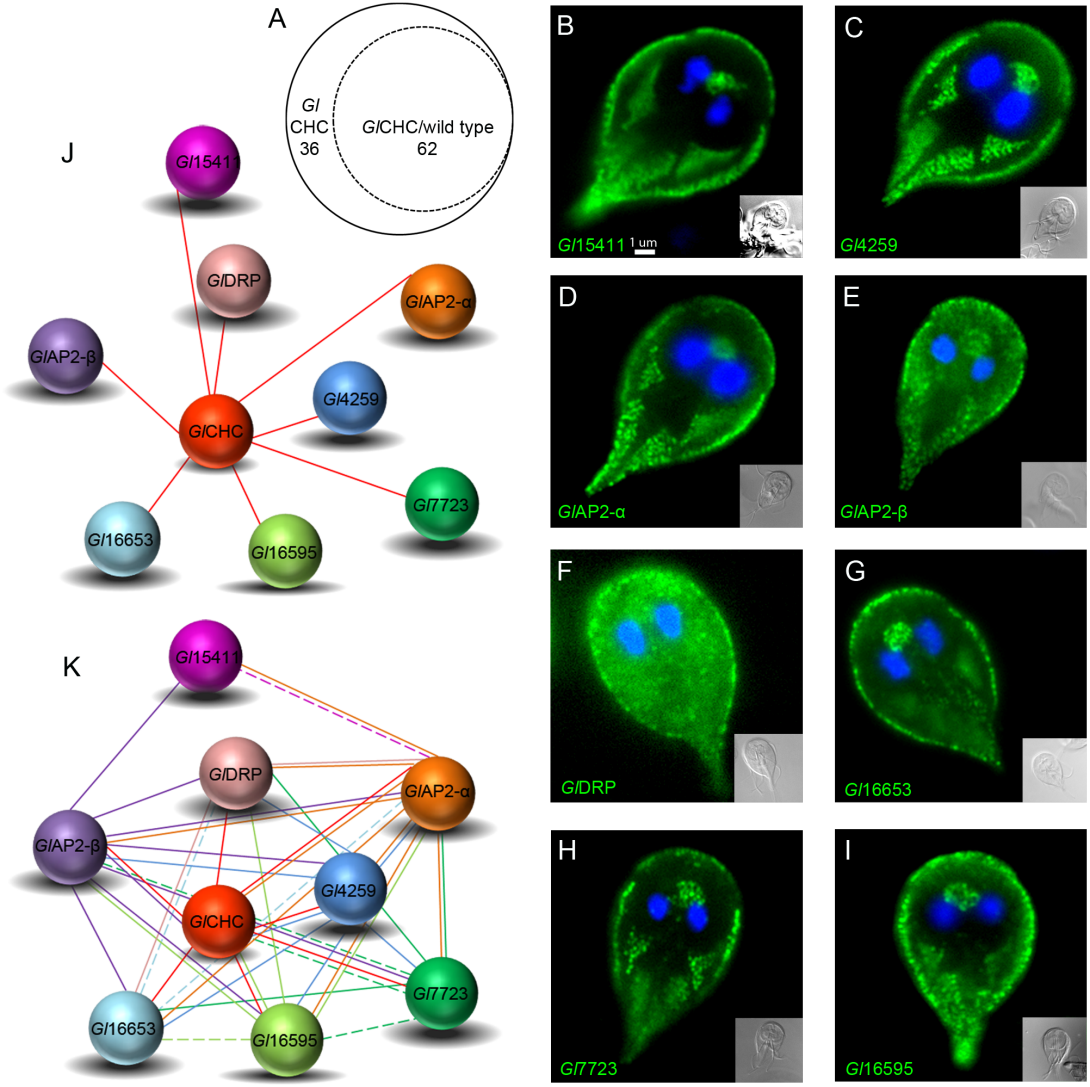
Proteomic and molecular analyses define GlCHC interacting proteins. (A) Venn diagram indicating 36 specific hits for the *Gl*CHC derived co-IP and 62 hits present in both co-IP datasets. (B-I) IFA validation of HA-tagged candidate *Gl*CHC interacting partners: aside from *Gl*DRP (F), all proteins exhibit exclusive localization in the cell cortex. Cells were imaged at maximum width, where nuclei and the bare-zone are at maximum diameter. Representative images for HA-tagged *Gl*15411(B), *Gl*4259 (C), *Gl*α-adaptin(D), *Gl*β-adaptin(E), *Gl*16653(G), *Gl*7723(H) and *Gl*16595(I). (J) *Gl*CHC-coIP based interactome validated by subcellular localization of reporter constructs. Each *Gl*CHC-interacting protein depicted in (J) was used in reverse coIP analyses (K) to further validate the interactions. Full and dashed lines indicate high and low interaction stringency parameters, respectively.

**Fig 9:**
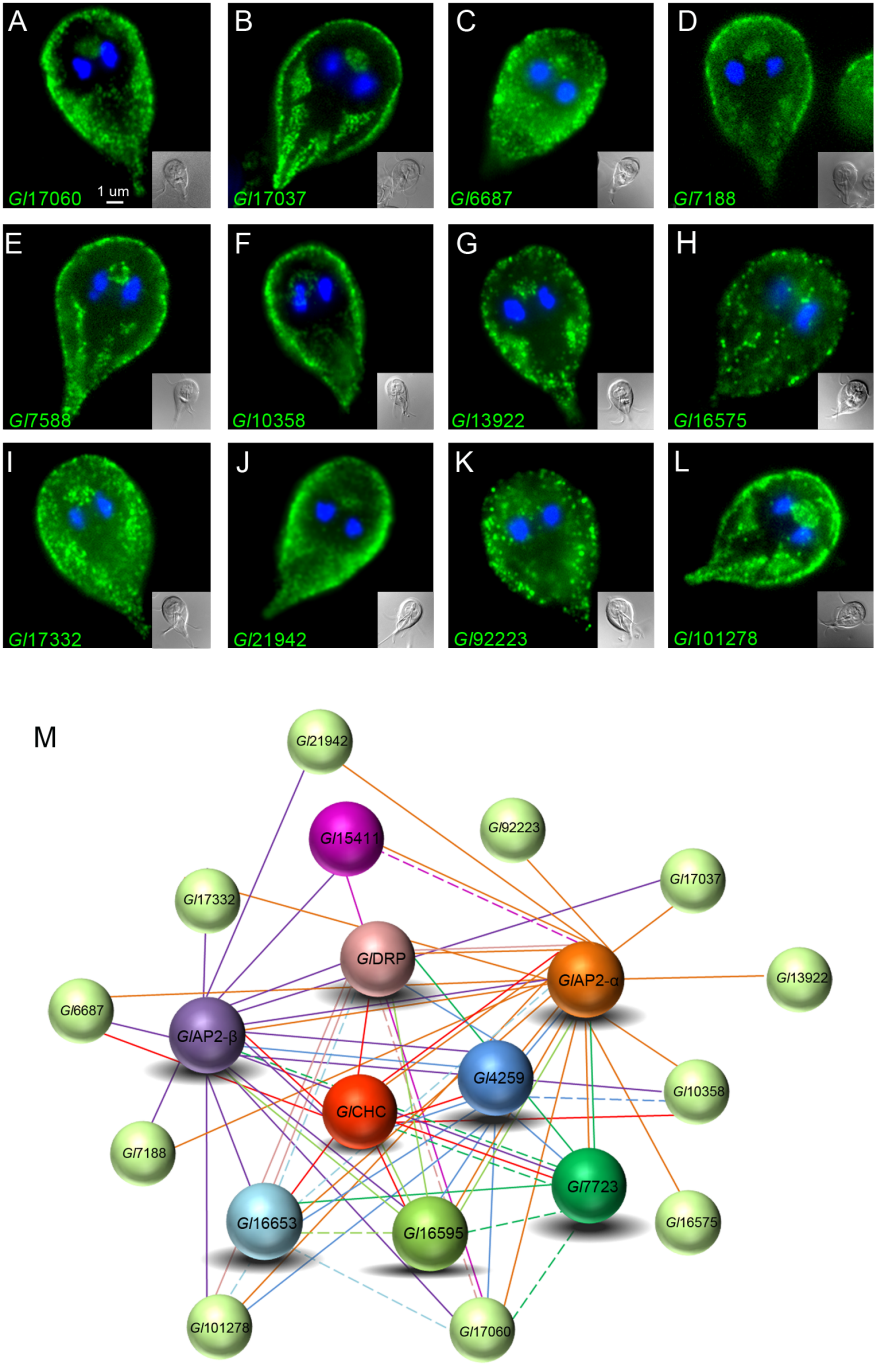
An expanded GlCHC-interactome. (A-L) IFA validation of HA-tagged candidate proteins obtained by reverse coIP: All reporter constructs localize, in varying degrees, to the cell cortex. Aside from 9 H and K, all reporters show deposition at the barezone, an indicator of PV-associated localization. Cells were imaged at maximum width, where nuclei and the bare-zone are at maximum diameter. Representative images for HA-tagged *Gl*17060(A), *Gl*17037(B), *Gl*6687(C), *Gl*7188*(D)*, *Gl*7588*(E)*, *Gl*10358*(F)*, *Gl*13922*(G)*, *Gl*16575*(H)*, *Gl*17332*(I)*, *Gl*21942*(J)*, *Gl*92223*(K) and*, *Gl*101278*(L)*. *(M)* Expanded *Gl*CHC-interactome including IFA-validated HA-tagged proteins from A-L (spheres in light green). Full and dashed lines indicate high and low interaction stringency parameters, respectively.

Filtration of the co-IP and ctrl. co-IP datasets in Scaffold4 (http://www.proteomesoftware.com/products/) using high stringency parameters (95_2_95, FDR 0%) and manual curation revealed 36 hits exclusive to the *Gl*CHC co-IP dataset. 62 proteins were identified in both datasets, albeit with different abundance (Fig 8A and S1 Table). We consistently detected *Gl*CHC in association to the three most important conserved endocytic factors, i.e. *Gl*DRP and the large α and β subunits of *Gl*AP2 (Fig 8B–8K). The co-IP experiment also retrieved *Gl*4295 in addition to identifying five novel, previously non-annotated clathrin interactors. These 8 predicted interactors were epitope-tagged and IFA analysis unambiguously localized all 8 to PVs at the cell cortex (Fig 8B–8I and S9 Fig). To validate these protein-protein interactions (Fig 8J) we performed reverse co-IP for each of the 8 candidate interaction partners. We defined the following stringent criteria for inclusion into the interactome model (Figs 8K and 9M): i) exclusive detection with ≥3 spectral counts in bait-specific datasets or ii) an enrichment of peptide counts ≥3 with respect to the ctrl. co-IP dataset (S1 Table). Full lines in the interactomes represent detection with high stringency parameters (95_2_95, FDR 0% in Scaffold) and dashed lines show detection with slightly relaxed stringency (95_2_50, FDR 0–0.8% in Scaffold). The 8 reverse co-IP experiments confirmed strong interaction of endogenous *Gl*CHC with all other bait proteins (Fig 8K) with the exception of *Gl*15411. This predicted metabolically inactive NEK kinase [58] appears to be associated only peripherally to assemblies and interacts with *Gl*CHC via the two large AP subunits. In contrast, α and β subunits of *Gl*AP2 are invariably associated to all other proteins in the interactome suggesting a key function in integrating the core *Gl*CHC assembly. From a phylogenetic point of view a central role for AP2 is not surprising given its hub function in clathrin networks in higher eukaryotes [54, 59]. The σ- and μ-adaptin subunits were not tagged for localization but were detected specifically in the co-IP datasets derived from *Gl*AP2 α and β subunits. This strongly suggests correct incorporation of the epitope-tagged large subunits into the heterotetrameric complex. Of note, 5 of the 8 validated *Gl*CHC-associated proteins harbor phosphoinositide-binding modules. The interaction of the AP2 protein complex with phosphatidylinositol 4,5-bisphosphate (PtdIns(4,5)P_2_) at the PM is well established in many eukaryotes [54]. The novel *Gl*CHC assembly factor *Gl*16653 harbors a FYVE (Fab1, YOTB/ZK632.12, Vac1, and EEA1) domain predicted to bind to PtdIns(3)P, a membrane lipid enriched in early endosomes, on the internal vesicles of multivesicular bodies, and on the yeast vacuole [60, 61]. *Gl*16653 had been identified earlier by BLAST searches and was shown to specifically bind to PtdIns(3)P [62]. Two *Gl*CHC-interacting proteins, encoded by ORFs 7723 and 16595 contain a conserved Phox (PX) domain at the C-terminus. PX domains mediate membrane-specific targeting of more than 40 mammalian proteins including the endocytosis-associated sorting nexins (SNX) [63] and are highly specific for phosphoinositides. Consistent with the position of *Gl*CHC assemblies at the interface of PM and PV membranes and the absence of membrane interaction modules on *Gl*CHC, this data also revealed three novel proteins that potentially link *Gl*CHC to intracellular membranes in addition to *Gl*AP2.

The filtered and manually curated MS datasets from all 9 co-IPs were used to extend the *Gl*CHC interactome by detecting overlaps. The datasets of α- and β- *Gl*AP2 subunits alone yielded 12 additional proteins whose epitope tagged variants localized to the cell cortex in transgenic cells by IFA (Fig 9A–9L). In total, the co-IP datasets yielded 32 candidate CHC assembly proteins of which 21 (65.62%) showed a corresponding subcellular distribution at the cell cortex (Figs 6A, 8B–8I and 9A–9L, S1 Table). These results validate our experimental approach for detecting weak and transient protein interactions such as the ones occurring in canonical clathrin coat assemblies [54].

Taken together, data generated in a series of co-IP experiments reveal a small but highly interconnected protein network in *Gl*CHC assemblies with *Gl*AP2 subunits as central organizers and a highly stable *Gl*CHC-*Gl*4295 structural complex. Not surprisingly, α-adaptin shows the most extended interactome, compared to other tested bait proteins, as in the more complex endocytic systems of higher eukaryotes [64–67]. In addition to underscoring the central role of the conserved factors *Gl*CHC, *Gl*AP2, and *Gl*DRP within *Gl*CHC assemblies, this analysis demonstrates the presence of additional monomeric membrane adaptor proteins and interaction with potential cargo receptors.

### Conserved endocytic factors *Gl*DRP and *Gl*AP2 have dynamic distributions

**Fig 10:**
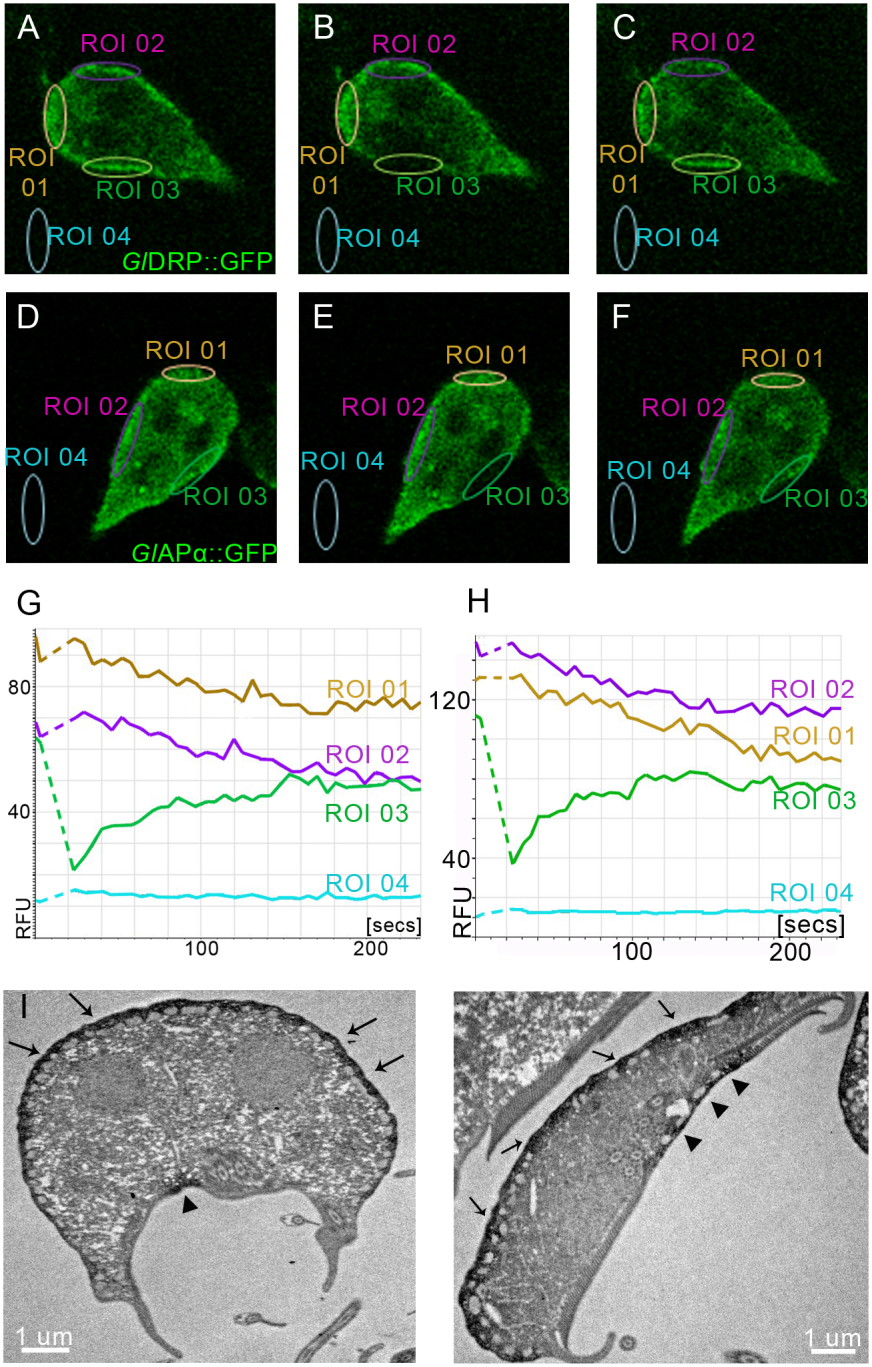
GFP-tagged GlDRP and GlAPα reporters show dynamic membrane association and the APEX2-2H-GlDRP reporter localizes in the cell cortex. (A-C,G) FRAP analysis of *Gl*DRP::GFP shows complete recovery after 200s for the bleached ROI03. Pre-bleach (A), post-bleach t0 (B), post-bleach t220 (C). (D-F, H) FRAP analysis of a GFP-tagged reporter for *Gl*α-adaptin::GFP shows complete recovery after 200s for the bleached ROI03. Pre-bleach (D), post-bleach t0 (E), post-bleach t220 (F). RFU: relative fluorescence units. (I) Representative examples of tEM images of APEX2-2HA-*Gl*DRP expressing cells after exposure to DAB for 5min show reporter localization in the PPI (arrows and arrow-heads). Note the distinct signal in the PPI of the bare-zone (arrow-heads).

The static nature of giardial clathrin assemblies at the PPI is consistent with the lack of turnover of *Gl*CHC and *Gl*4295 at membranes (Figs 5 and 6). In addition, the absence of motifs and machinery for disassembly of these structures points to an alternative, as yet unknown, mechanism for turning over of CHC assemblies. Nevertheless, the identification of the conserved components *Gl*AP2 and *Gl*DRP as interactors of *Gl*CHC and *Gl*4295 suggested that some factors of the CHC core interactome may have dynamic turnover, e.g. in a model where *Gl*CHC and *Gl*4295 constitute a static platform surrounded by mobile partners. To test this, we performed FRAP analysis of corresponding GFP fusions for both *Gl*AP2α and *Gl*DRP in transgenic cells. Identical conditions for live-cell confocal microscopy revealed rapid (≤150 sec) fluorescence recovery for both reporters (Fig 10A–10H), in line with their dynamics in higher eukaryotes [64, 65]. Compared to data for *Gl*CHC and *Gl*4295 (Figs 5a and 6h), the significant difference in turnover for GFP-tagged *Gl*AP2α and *Gl*DRP predicted a more peripheral distribution for these proteins at *Gl*CHC assemblies. Although the *Gl*DRP::APEX signal in tEM was strictly restricted to the region between the PM and the adjacent PV membrane it appeared more dispersed compared with APEX-tagged *Gl*CHC and *Gl*4295 variants (Fig 10I), which was consistent with transient recruitment of this large GTPase [64]. For as yet unknown reasons no signal for the *Gl*AP2::APEX reporter could be obtained, although this construct could be clearly detected in IFA by labelling of the HA epitope tag (S4D Fig). However, the μ subunit of the *Gl*AP2 was previously localized at the PPI by immunoelectron microscopy [19]. Taken together, these data provide further evidence that both static and dynamic core interactome components of *Gl*CHC assemblies function in the narrow zone of the PPI.

## Discussion

### Cell polarization and remodeling of giardial endocytic organelles

*Giardia* is the only diplomonad with an organelle that allows mechanical attachment to the host small intestinal epithelium to prevent expulsion by peristalsis [68]. Hence, the ventral disk with its complex cytoskeleton architecture is, in evolutionary terms, a new invention in this lineage, which resulted in a distinct dorso-ventral polarization of the parasite. Exposure of the dorsal PM to the gut lumen was likely the main driver of a subcellular reorganization of the endocytic system in close proximity with this membrane. Nevertheless, a population of PVs underlies the PM at the bare zone at the center of the ventral disk. *Spironucleus* spp., the closest relatives of *Giardia* [69], ingest fluid phase matter via a single cytostome into food vacuoles (S10 Fig). This bulk endocytic system with a linearly organized digestive process is typical for ciliates but occurs also in other protozoa with completely different life styles (e.g. *Plasmodium*) [11, 70, 71]. Limiting endocytosis to a single protected site may be particularly advantageous for parasitic protozoa with dense surface coats that are permanently exposed to host immune effectors and/or deleterious substances. A case in point is the flagellar pocket of trypanosomes, an evolutionary adaptation that allows clathrin-mediated endocytosis in a hostile environment [72]. Although *Giardia* meets all those criteria, its endocytic organelle system is maximally dispersed, occupying all exposed sites at the PM of attached trophozoites. We hypothesized that this unique adaptive design entailed significant molecular changes in the endocytic transport machinery. Of particular interest was the function of clathrin given the extremely short distances between PVM and PM, and the absence of CCVs in tEM micrographs [73]. Consistent with this observation, the arrangement of PVs appears to be designed for rapid and efficient sampling of the extracellular space.

### Giardial clathrin as a non-dynamic component of static focal assemblies

Continuous sampling of the environment by trophozoites was demonstrated previously in experiments with fluid-phase markers [3, 38]. The subcellular localization of PVs was fixed and EM micrographs consistently identified direct connections of PM and PV membranes (this study, [4]) as well as occasional direct links of both to the ER (S11 Fig). This is consistent with previous reports of some fluid phase material being transported via PVs directly into the ER [38]. Taken together, this raises fundamental questions about the organization and molecular underpinnings of fluid phase and receptor-mediated endocytosis in *Giardia*. In the present study we focused on the function of *Gl*CHC and its interactions with other components of focal assemblies detected with a highly specific polyclonal antibody [3, 19, 38, 74]. Ectopically expressed, epitope- or GFP-tagged *Gl*CHC variants localized specifically to *Gl*CHC assemblies by fluorescence microscopy, with no indications of a significant cytoplasmic pool. Imaging of *Gl*CHC assemblies by STED microscopy indicated a size consistent with focal assemblies (~50nm) rather than membrane coats or sheets associated with the PM or PV membranes. Typically, CCVs measure 80-150nm [11, 39–41] whereas flat clathrin sheets present on the cytosolic PM-leaflet in some mammalian cells are often >500nm in size [15, 16, 75–77]. In line with previous approaches to measure clathrin turnover during CCV formation, we quantified fluorescence recovery of photobleached *Gl*CHC::GFP or *Gl*4295::GFP reporters and measured the longevity of CHC assemblies. Membrane-associated triskelia show frequent exchange of CHC::GFP reporters in FRAP experiments with recovery times of <20s [78]. Unexpectedly, our data unequivocally showed that turnover of both reporters at assemblies in living cells was not measurable within a time frame of > 10min. Recruitment and assembly of CHC at membranes during canonical CME is highly dynamic and typical CCV lifetimes range between 30-60s [79]. FRAP based recovery values for fluorescently tagged clathrin light chains, a robust approach to study the dynamics of membrane-associated clathrin triskelia [80], are as fast as 5-10s [14] whilst the larger CHC plaques exhibit lifetimes sometimes exceeding 600s but with 50% reporter molecule turnover in in bleached areas after 115s [77]. FRAP analysis of *Gl*CHC::GFP and *Gl*4295::GFP dynamics in this study were in complete agreement and suggested high stability as well as extended lifetimes of *Gl*CHC assemblies. Taking into account that live-cell GFP-based imaging of assemblies is challenging in microaerophilic conditions, this exceeds the duration of complete CME events or the lifetimes of CHC plaques by far [77, 79, 80]. Of note, careful analysis of the tracking experiments provided no indication for a separate population of *Gl*CHC assemblies in the cytosol, e.g. coated vesicles derived from a *trans* Golgi compartment. This is consistent with the lack of a Golgi apparatus in proliferating trophozoites [8, 74] and the absence of any hits for AP1 subunits in *Gl*CHC co-IP datasets.

GFP is considerably larger than any epitope tag and may therefore affect functionality of its fusion partner(s) [81]. Transgenic *G*. *lamblia* cells constitutively expressing GFP fusions to *Gl*CHC, *Gl*4259, *Gl*DRP and *Gl*APα were indistinguishable from untransfected control cells and from transgenic cells expressing corresponding epitope-tagged variants (S4A–S4D Fig, Figs 5–8 and 10). Similarly, the subcellular distribution of all GFP fusion reporters was identical to the distribution of the corresponding HA-tagged variants. Specifically for *Gl*CHC, endogenous, HA and GFP -tagged variants were invariably detected in close proximity to PVs, with no indication of mislocalization for the recombinant variants. Although it is not currently feasible to test functionality by complementing *Gl*CHC knock-down (this work) or knock-out lines, these data strongly support the notion that GFP- and HA- tagged variants for *Gl*CHC, *Gl*4259, *Gl*DRP and *Gl*APα are correctly incorporated into clathrin assemblies and are likely functional.

Taken together, this is direct evidence for the highly stable nature of *Gl*CHC assemblies and strongly suggests that giardial clathrin, unlike other well-characterized homologues [72, 82–85], is not part of a dynamic process involving formation of short-lived membrane carriers for vesicular transport.

### What drives and maintains clathrin recruitment to membranes in *G*. *lamblia*?

Formation of static focal assemblies by *Gl*CHC is in line with the observed lack of coated pits and CCVs in trophozoites. However, this raises the question how giardial clathrin is recruited to membranes. In well-described systems dynamic recruitment of clathrin triskelia to the cytoplasmic face of the PM is mediated by AP2. Specific domains of the two large subunits interact with transmembrane cargo proteins or phospholipid headgroups [86]. A recent single molecule study in BSC1 cells showed recruitment of one clathrin triskelion by 2 membrane bound AP2 complexes or 2 by 4, respectively, and demonstrated the importance of the triskelia to stabilize the initiating complexes allowing further progression of recruitment [87]. Furthermore, monomeric adapter-like stonins, GGAs or Dab2 increase the spectrum of cargo molecules that can be recognized and further stabilize association of clathrin to the PM [10]. However, with the exception of AP2, none of the common components associated with recruitment of clathrin to membranes are conserved in *Giardia*.

Interestingly, despite the longevity of *Gl*CHC assemblies, AP2α::GFP which localizes both to PM and PV membranes at PPIs (therefore to both poles of *Gl*CHC assemblies) [19] shows considerable turnover and recovery in ~160 seconds in FRAP experiments. Canonical AP2 is excluded from established membrane coats of coated pits, plaques and CCVs and acts only in zones of triskelion recruitment at the edges of clathrin coats [67, 88, 89]. Given the stability and longevity of assemblies built from tightly interconnected *Gl*CHC and *Gl*4259, the observation that these structures are not subject to ordered dismantling is contrasted by turnover of *Gl*AP2 in FRAP experiments. Taken together with the apparent lack of *Gl*CHC and *Gl*4259 cytoplasmic pools, the numerous interactions of *Gl*AP2 with other components of clathrin assemblies suggest that *Gl*AP2 acts at the edges of these long-lived structures. This hypothesis is also consistent with the identification of *Gl*AP2 and *Gl*DRP associated to *Gl*CHC only when co-IP experiments were performed in cross-linking conditions. In the absence of a recruitment and disassembly cycle we hypothesize that the role of *Gl*AP2 is geared towards stabilizing the connection between the rigid structure of the assembly proper and the flexible membranes at either end. Constant turnover of *Gl*AP2 at the edges of assemblies may contribute to mechanisms that keep these structures positioned at the PPI. Recent experiments in cell-free systems highlighted a switch function of *Gl*AP2 linked to interaction with PtdIns(4,5)P2 and transmembrane cargo proteins and the requirement for transmembrane proteins containing di-leucine ([DE]XXXL[LI]) [90] or tyrosine-based (YXXΦ) [91] endocytic motifs in their cytoplasmic C-termini [92]. Indeed, co-IP experiments with *Gl*AP2 subunits (Fig 9) identified 2 related type Ia transmembrane proteins; ORFs *Gl*7188 (1103aa) and *Gl*13922 (1087aa), the first carrying a canonical YXXΦ motif (residues 1090–1093; Tyr-Leu-Arg-Val) in the predicted cytoplasmic tail. The presence of an endocytosis motif in *Gl*7188 suggests functional receptor-mediated endocytosis as previously demonstrated for the giardial protease ESCP [93]. However, in the absence of canonical CME in *Giardia* the exact mechanism for *Gl*7188 uptake into endocytic compartments requires further investigation. This putative receptor is part of the *Gl*AP2 interactome but is not pulled down by either *Gl*CHC or *Gl*DRP. Hence, *Gl*AP2 and associated proteins at the rims of *Gl*CHC assemblies are likely dynamic elements which recruit and organize transmembrane and cytoplasmic factors to stabilize the PPI as a whole. *Gl*AP2 cycles between a cytosolic and a membrane-bound state and likely undergoes the same conformational changes as canonical AP2 as it binds to endocytic motifs in C-termini of transmembrane proteins and to phospholipid headgroups [86]. However, maintaining the physical link between membranes and *Gl*CHC assemblies likely requires additional factors with lipid binding domains.

### Phospholipid binding proteins in *Gl*CHC assemblies

Electron microscopy frequently shows deep invaginations of the PM towards PV membranes, with the membranes occasionally forming continuities. Although morphologically similar to canonical coated pits albeit smaller, these invaginations exhibit considerable membrane curvature. Interestingly, the *Gl*CHC core interactome revealed 3 proteins with predicted phosphatidylinositol binding domains. The single giardial FYVE domain protein (*Gl*16653) has confirmed PdtIns3P binding specificity [62]; two PX-domain proteins could act as monomeric adaptor proteins. However, the latter lack BAR domains and their involvement, if any, in generating membrane curvature cannot be predicted from sequence alone. In canonical systems PX-domain proteins such as sorting nexins form complexes with dynamin-2 and bind to PM-associated AP2 molecules lining developing coated pits [94]. Our data show robust interactions of these three phosphoinositide (PI)-binding proteins with the *Gl*CHC network. Further investigation is required to determine their possible involvement in selective recruitment of transmembrane proteins as shown in other systems [10, 95, 96] or a role in the morphogenesis and stabilization of *Gl*CHC assemblies. The giardial genome codes for 6 PX domain proteins. All members of this completely species-specific class of proteins localize to PVs and appear to contribute significantly to organizing PV membrane associated proteins, but only 2 are part of the extended *Gl*CHC interactome. In addition to their selective interaction with *Gl*CHC assemblies, the 6 members have PIs binding profiles with different specificities, and N-terminally tagged variants have distinct distributions at PV membranes (Zumthor, Cernikova & Hehl, in preparation).

Taken together, analysis of conserved and novel *Gl*CHC interacting partners reveals two functionally distinct groups with respect to dynamics of association with membranes: at least two highly static structural elements, *Gl*CHC and a putative light chain *Gl*4259, and dynamic components *Gl*AP2 [19] and *Gl*DRP [3] with turnovers at membranes similar to orthologues in coated pits of higher eukaryotes [64, 65]. Interestingly, the extended interactome accurately reflects this unique dichotomy and is conceptually in line with the absence of conserved disassembly factors HSC70 and auxillin [97] in *G*. *lamblia*. Such a striking divergence from canonical CME systems can also be appreciated in other parasitic protozoans such as *T*. *brucei*. CME in trypanosomes occurs in the absence of AP2 [98] and is mediated by a cohort of trypanosome-specific clathrin-associated proteins [99]. Similar to *Giardia*, trypanosomes employ a mixture of conserved and unique components to achieve a conserved function i.e. uptake from the extracellular space, using evolutionarily and molecularly distinct processes [100].

**Fig 11:**
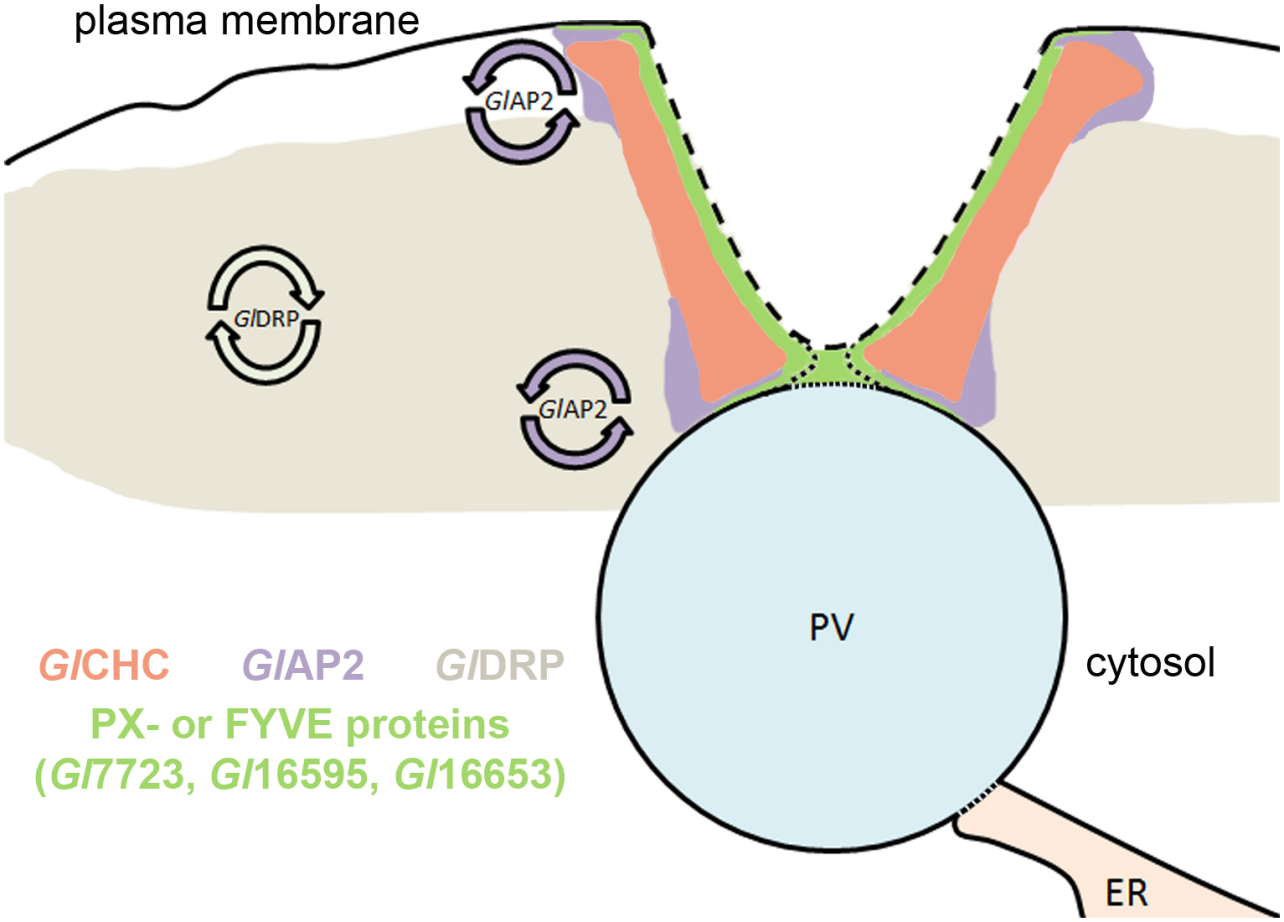
A working model for the organization of clathrin and associated proteins at the PPI. The model shows a cross section through the PPI including a PV organelle and the PM. For endocytic uptake, PM invaginations fuse with PV membranes to create luminal continuity with the extracellular environment. This is the most likely explanation for bulk uptake of fluid phase markers and occasional direct transport to the ER [3, 38]. After uptake, membranes are separated whilst clathrin assemblies remain at the PPI. The distribution of relevant proteins is based on experimental data and functional properties [19]. Dashed lines indicate where membrane remodeling may occur. Circular arrows designate dynamic protein properties.

Our and previous data support a working model (Fig 11) for clathrin assemblies at the interface of PV-PM membranes, in which access to the fluid extracellular environment in the host’s gut lumen, is achieved when the invaginated PM fuses with the PV. We have visualized this as replacement of fluid phase markers in the PV lumen by a combined endocytic/exocytic event [3]. Whether this phenomenon is always receptor-based and highly regulated or mainly spontaneous and stochastic remains an open question and probably depends on the nature of the cargo (membrane-associated *vs*. soluble). The most likely scenario calls for PM invaginations stabilized by *Gl*CHC-*Gl*4259 scaffolds. These are rimmed by dynamic *Gl*AP2 and linked to membranes by lipid binding proteins such as PX- and FYVE-domain proteins (akin to “frozen” clathrin coated pits). In canonical CME, coated pit formation can be driven by actin-mediated deformation of the plasma membrane [101]. Although previous reports established a link between actin and endocytosis in *Giardia* [102], our co-IP experiments did not detect any interaction between clathrin assemblies and actin.

Given the longevity of giardial clathrin assemblies we hypothesize that they are not formed *de novo* for each endocytic event but stabilize PM invaginations during multiple rounds. Successful uptake requires controlled membrane fusion at the interface of PM invaginations and PV membranes. The regulatory aspects of this process remain unclear. However, given its association to clathrin assemblies and its role in PV morphogenesis [3], the single dynamin in *Giardia* may be a key enzyme in membrane remodeling processes. Thus, the dispersion of the endocytic system with a large number of independently regulated points of entry allowing direct communication with the extracellular space may be an ideal strategy for *Giardia* trophozoites to thrive in the changing environment of the small intestine. The PV system under the protective layer of the giardial surface coat could act as a safety lock, allowing mostly unselective entry of fluid phase material for digestion and selective further endocytic transport to the ER lumen [38]. At the same time indigestible or potentially harmful substances could be contained safely within PVs until elimination in the next round of “kiss and flush”.

## Supporting Information

S1 Table Oligonucleotides used in the study. (XLSX)

S2 Table Overview of all analyzed open reading frames identified by co-IP. (XLSX)

S1 Fig Confocal microscopy and image analysis of cholera toxin endocytosis. A) Representative volume images of cells (three dimensional reconstructions of image stacks) at 4 time points (10–60 min) post labeling with dextran-Oregon Green (fluid phase marker, green) and cholera toxin-AF594 (membrane marker, red). Nuclear DNA is labeled with DAPI (blue); scale bars: 2 μm. Insets: two dimensional scatter plots showing signal distribution (green, red) in voxels. B) Quantification of signal overlap: statistical analysis of Mander’s coefficients from five image sets per time point (each set containing an average of 3 cells). C) Enlarged volume images showing typical signal distribution at 10 and 60 min post labeling. Enlarged areas are indicated; scale bars: 2 μm.
(TIF)

S2 Fig Vector map for pPACV_Integ modified. (TIF)

S3 Fig Compilation of electron micrographs showing PV-associated PM-invaginations. (A-K) TEM images, (L-O) FIB-SEM images.
(TIF)

S4 Fig Localization and dynamics of GlCHC and interaction partners. Confirmation of the cortical localization of the APEX2 variants used in TEM by IFAs directed against the double HA-tags of (A) *Gl*4259 (B) *G*lCHC, (C) *Gl*DRP and (D) *Gl*AP2-alpha. (E) No recovery is measured after >15 mins imaging of photobleached areas (ROI 1) in cells expressing a *Gl*CHC-GFP reporter. (F) Distribution of *Gl*CHC in cells constitutively expressing a *Gl*CHC-hub fragment. Scale bar: 10 μm.
(TIF)

S5 Fig The giardial clathrin heavy chain lacks the QLMLT motif essential for uncoating. ClustalW alignment of the C-terminal ends of clathrin heavy chains harboring conserved QLMLT motifs (red) with clathrin heavy chains from *G*. *lamblia* and its close relative *S*. *salmonicida*. Note how the giardial sequence ends just before the QLMLT motif. Conserved residues in green indicate robust sequence alignment upstream of the uncoating motif.
(TIF)

S6 Fig Subcellular distribution of the HA-tagged GlCHC variant in the cell line used for co-IP. A representative wide-field microscopy image shows reproducible *Gl*CHC-HA signals in transgenic trophozoites.
(TIF)

S7 Fig Structural overlap of iTASSER de novo predictions for Gl4259 and annotated clathrin light chains. Overlap of predicted structures for *Gl*4259 and annotated clathrin light chains from with (A) *T*. *reesei*, (B) *T*. *gondii*, (C) *S*. *cerevisiae*, (D) *M*. *musculus*, (E) *C*. *reinhardtii*, (F) *D*. *melanoogaster* and (G) *C*. *elegans*. (H) The table summarizes C-scores for all five iTASSER models predicted for each sequence. The models that were chosen for structural comparison are highlighted in green.
(TIF)

S8 Fig Titration of DSP used for co-IP assays with limited crosslinking. Immuno-detection (Western blot) of the GlCHC-HA reporter presents a shift from the monomeric form to higher molecular weight complexes, with increasing concentrations of DSP (0–3 mM). Molecular size (kDa) marker bands are indicated on the left.
(TIF)

S9 Fig Confocal microscopy and signal overlap analysis of novel GlCHC interacting partners and GlCHC. Immunofluorescence assays and confocal microscopy analysis of HA-tagged reporter lines for ORFs 15411, 7723, 16595, 16653, 10358, and 6687 (A–F), labeled for both the HA tag (green) and endogenous *Gl*CHC (red). Insets: two dimensional scatter plots showing signal overlap (green, red) in voxels. Nuclei are labelled with DAPI (blue).
(TIF)

S10 Fig Dextran-TxR uptake in S. vortens. (A) The fluid phase marker dextran-TxR is taken up into intracellular compartments of varying sizes. (B) Inset of (A) as indicated by the arrows. Insets: DIC images.
(TIF)

S11 Fig TEM micrographs of direct continuity of the ER with PVs (arrows) and the PM (arrow head). (TIF)
