# Supplementary figures and images for: Static Clathrin Assemblies at the Peripheral Vacuole—Plasma Membrane Interface of the Parasitic Protozoan Giardia lamblia
Source: PLoS Pathog. 2016 Jul 20;12(7):e1005756. doi: 10.1371/journal.ppat.1005756 (PMC4954726; doi:10.1371/journal.ppat.1005756)

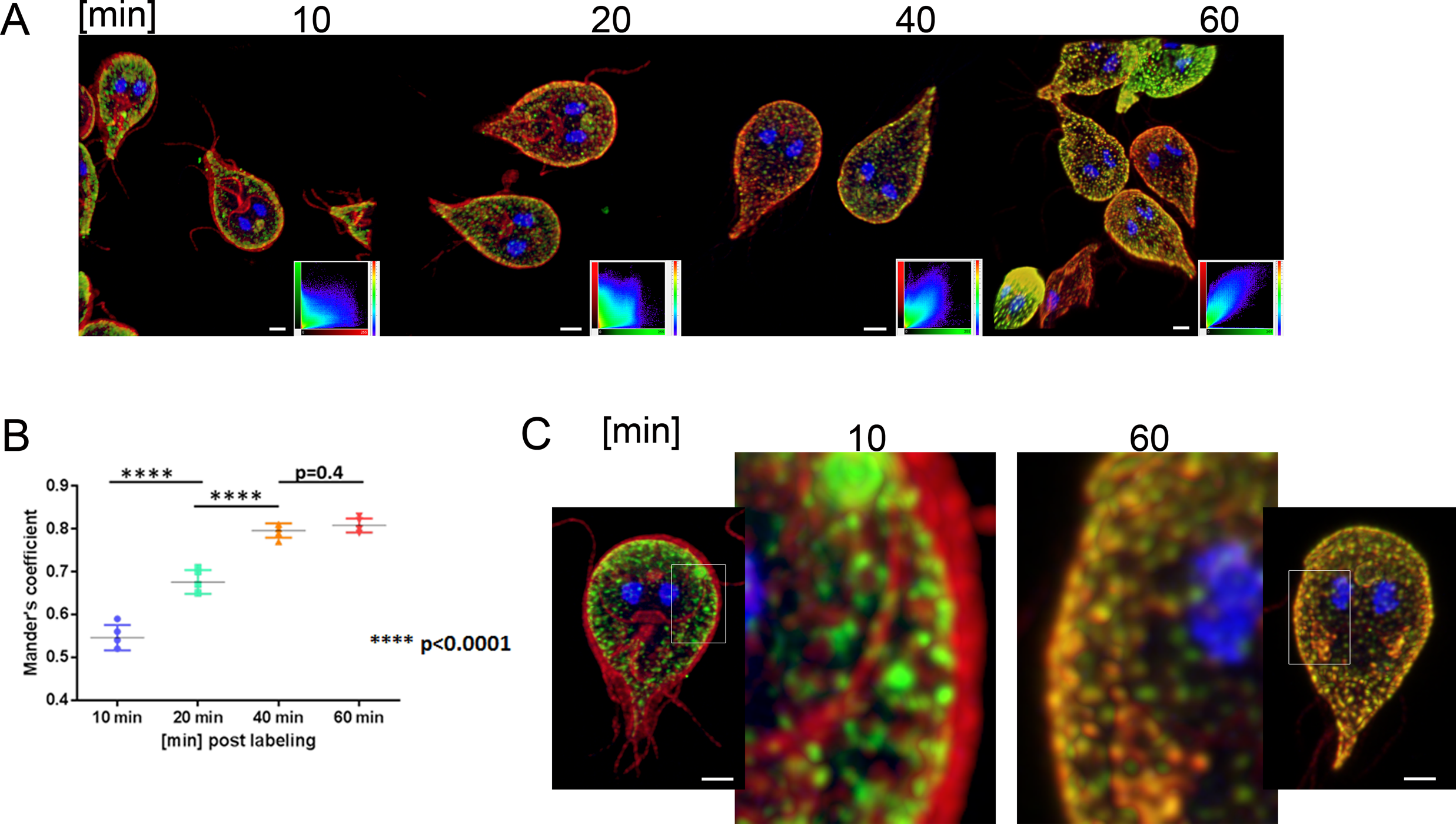

Supplement: S1 Fig — A) Representative volume images of cells (three dimensional reconstructions of image stacks) at 4 time points (10–60 min) post labeling with dextran-Oregon Green (fluid phase marker, green) and cholera toxin-AF594 (membrane marker, red). Nuclear DNA is labeled with DAPI (blue); scale bars: 2 μm. Insets: two dimensional scatter plots showing signal distribution (green, red) in voxels. B) Quantification of signal overlap: statistical analysis of Mander’s coefficients from five image sets per time point (each set containing an average of 3 cells). C) Enlarged volume images showing typical signal distribution at 10 and 60 min post labeling. Enlarged areas are indicated; scale bars: 2 μm. (TIF) [file ppat.1005756.s003.tif]

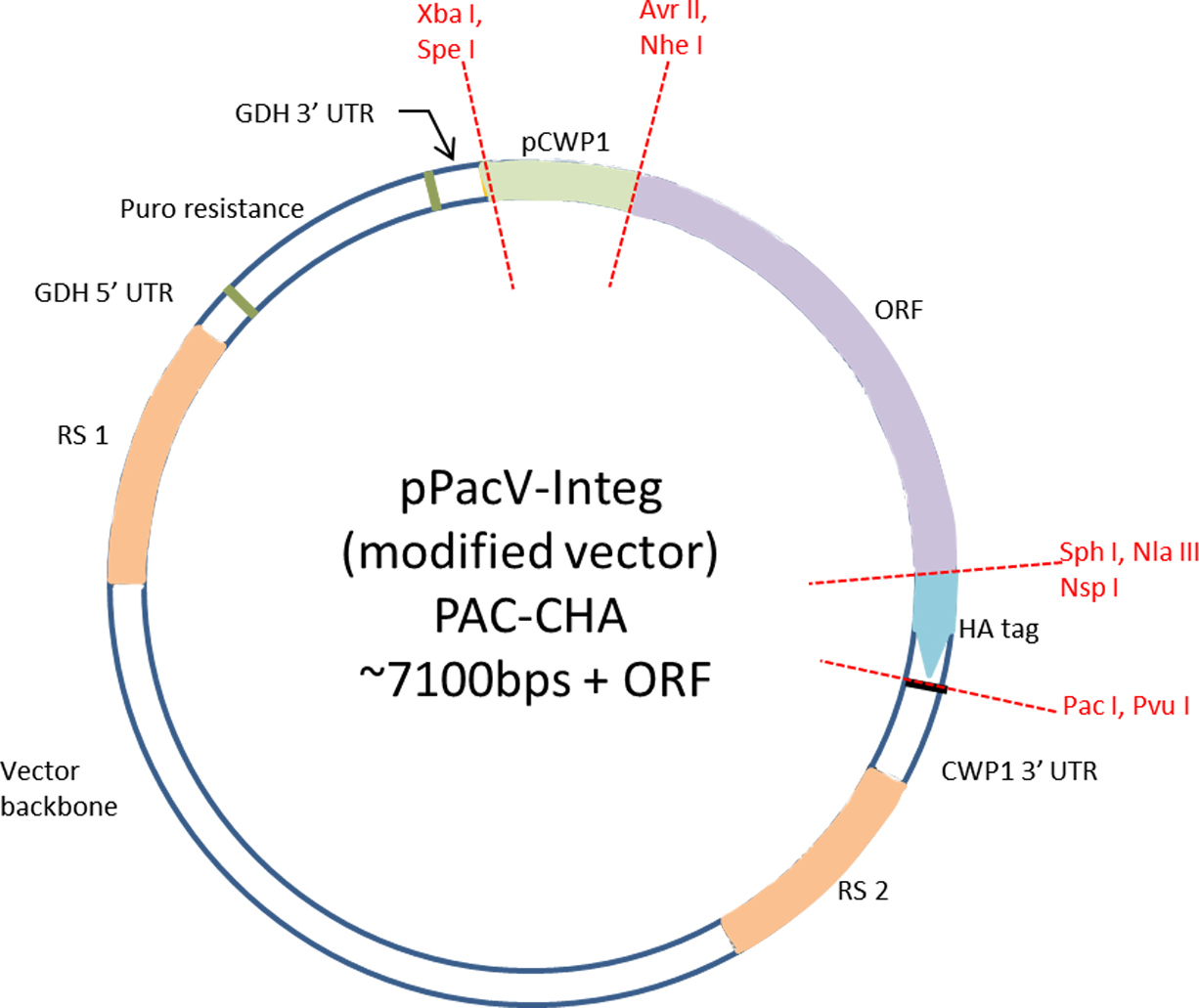

Supplement: S2 Fig — (TIF) [file ppat.1005756.s004.tif]

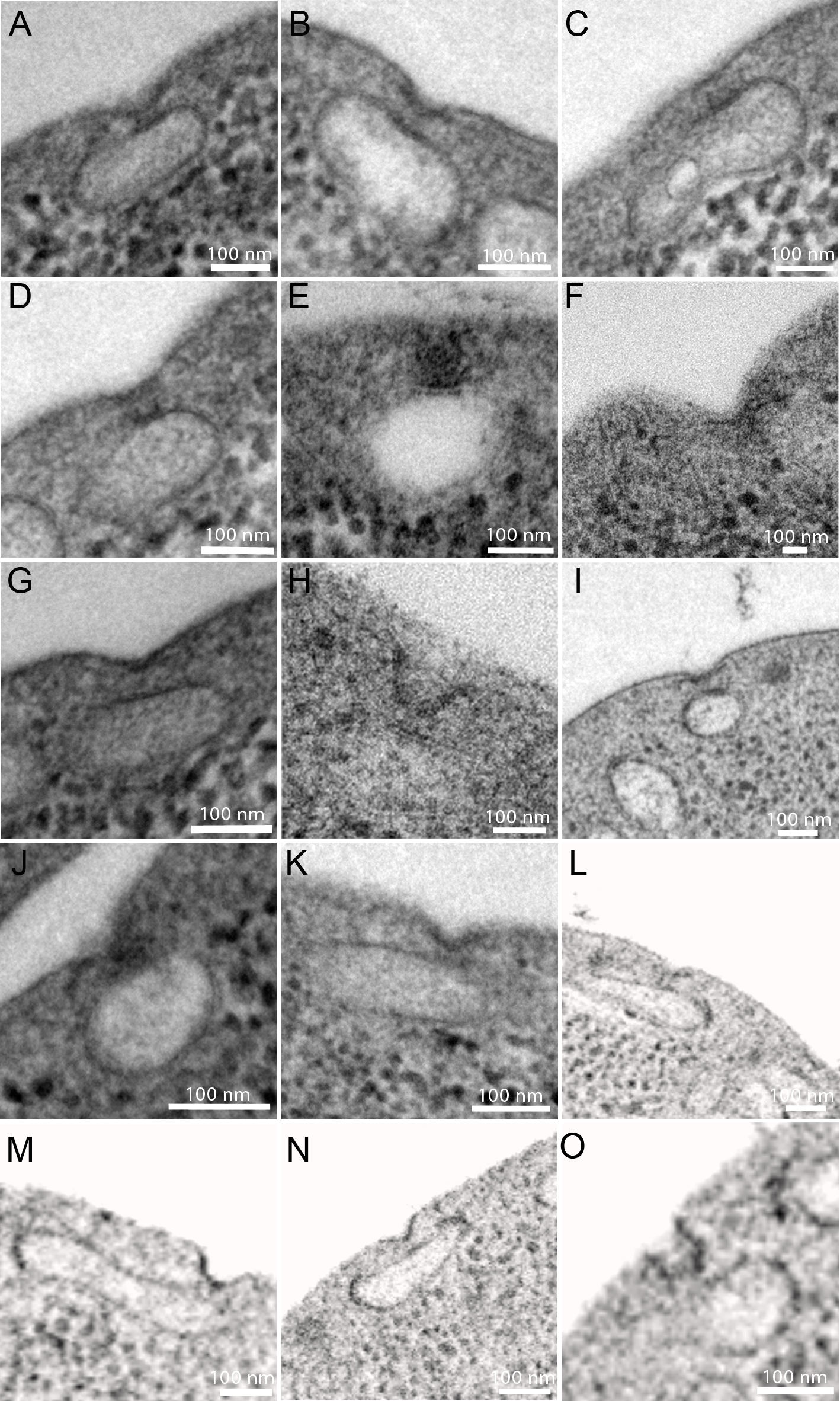

Supplement: S3 Fig — (A-K) TEM images, (L-O) FIB-SEM images. (TIF) [file ppat.1005756.s005.tif]

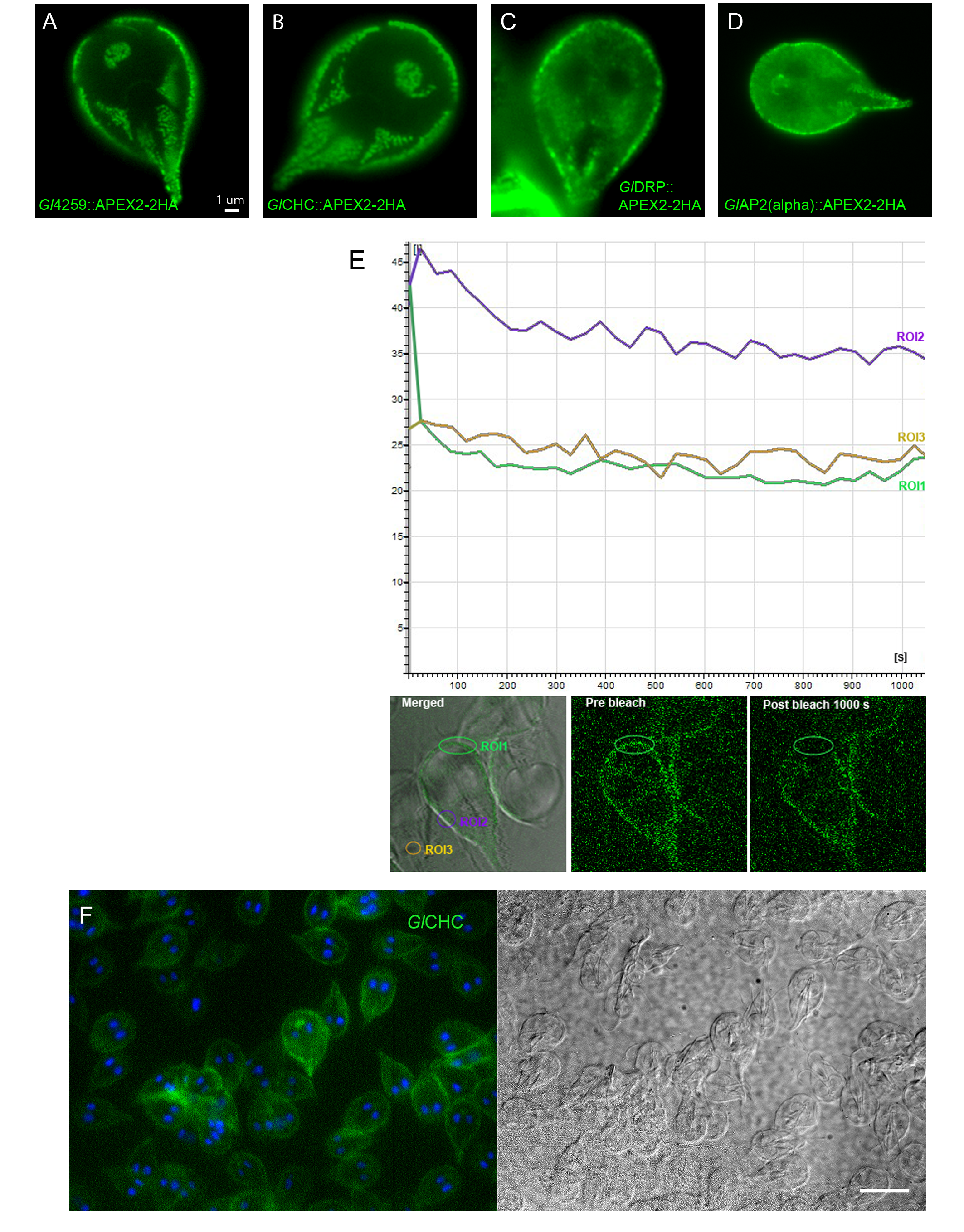

Supplement: S4 Fig — Confirmation of the cortical localization of the APEX2 variants used in TEM by IFAs directed against the double HA-tags of (A) Gl4259 (B) GlCHC, (C) GlDRP and (D) GlAP2-alpha. (E) No recovery is measured after >15 mins imaging of photobleached areas (ROI 1) in cells expressing a GlCHC-GFP reporter. (F) Distribution of GlCHC in cells constitutively expressing a GlCHC-hub fragment. Scale bar: 10 μm. (TIF) [file ppat.1005756.s006.tif]

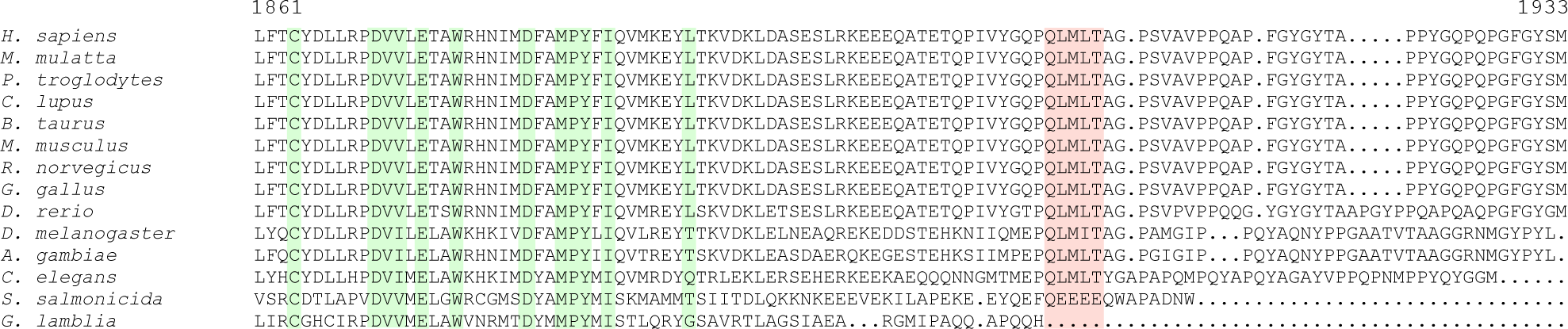

Supplement: S5 Fig — ClustalW alignment of the C-terminal ends of clathrin heavy chains harboring conserved QLMLT motifs (red) with clathrin heavy chains from G. lamblia and its close relative S. salmonicida. Note how the giardial sequence ends just before the QLMLT motif. Conserved residues in green indicate robust sequence alignment upstream of the uncoating motif. (TIF) [file ppat.1005756.s007.tif]

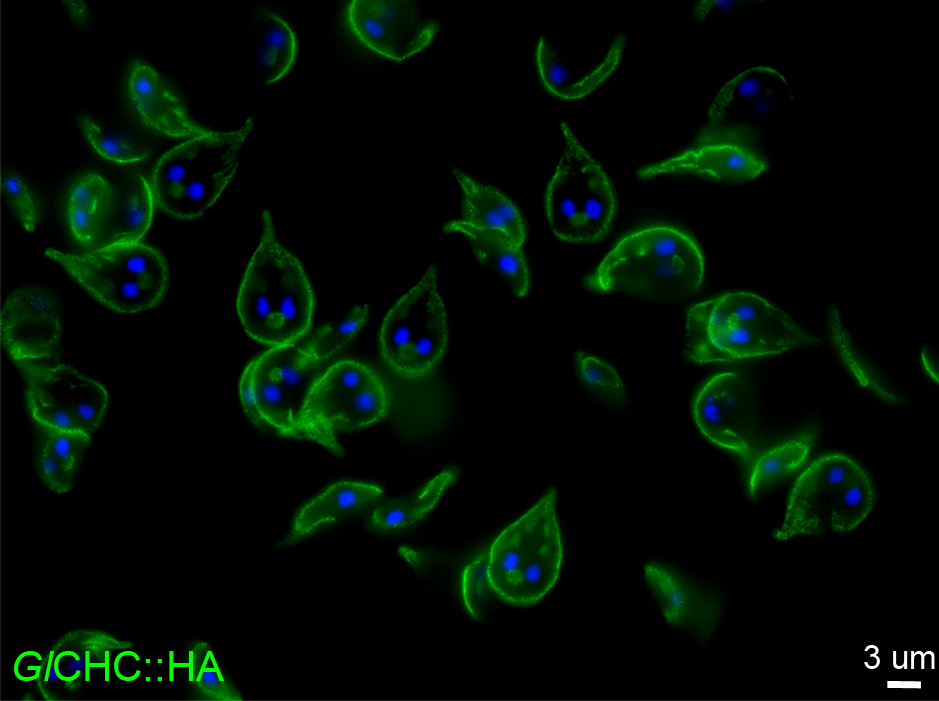

Supplement: S6 Fig — A representative wide-field microscopy image shows reproducible GlCHC-HA signals in transgenic trophozoites. (TIF) [file ppat.1005756.s008.tif]

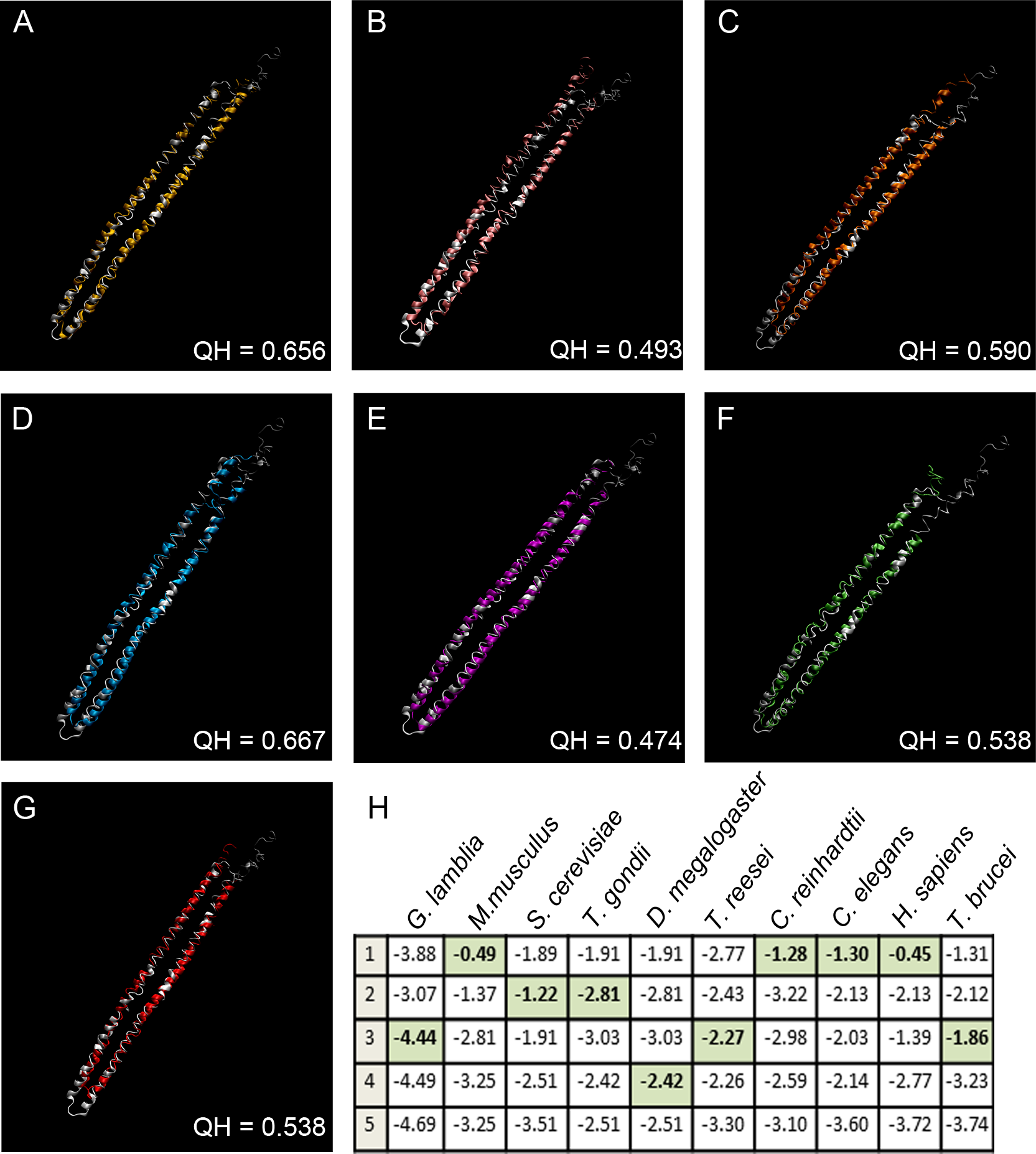

Supplement: S7 Fig — Overlap of predicted structures for Gl4259 and annotated clathrin light chains from with (A) T. reesei, (B) T. gondii, (C) S. cerevisiae, (D) M. musculus, (E) C. reinhardtii, (F) D. melanoogaster and (G) C. elegans. (H) The table summarizes C-scores for all five iTASSER models predicted for each sequence. The models that were chosen for structural comparison are highlighted in green. (TIF) [file ppat.1005756.s009.tif]

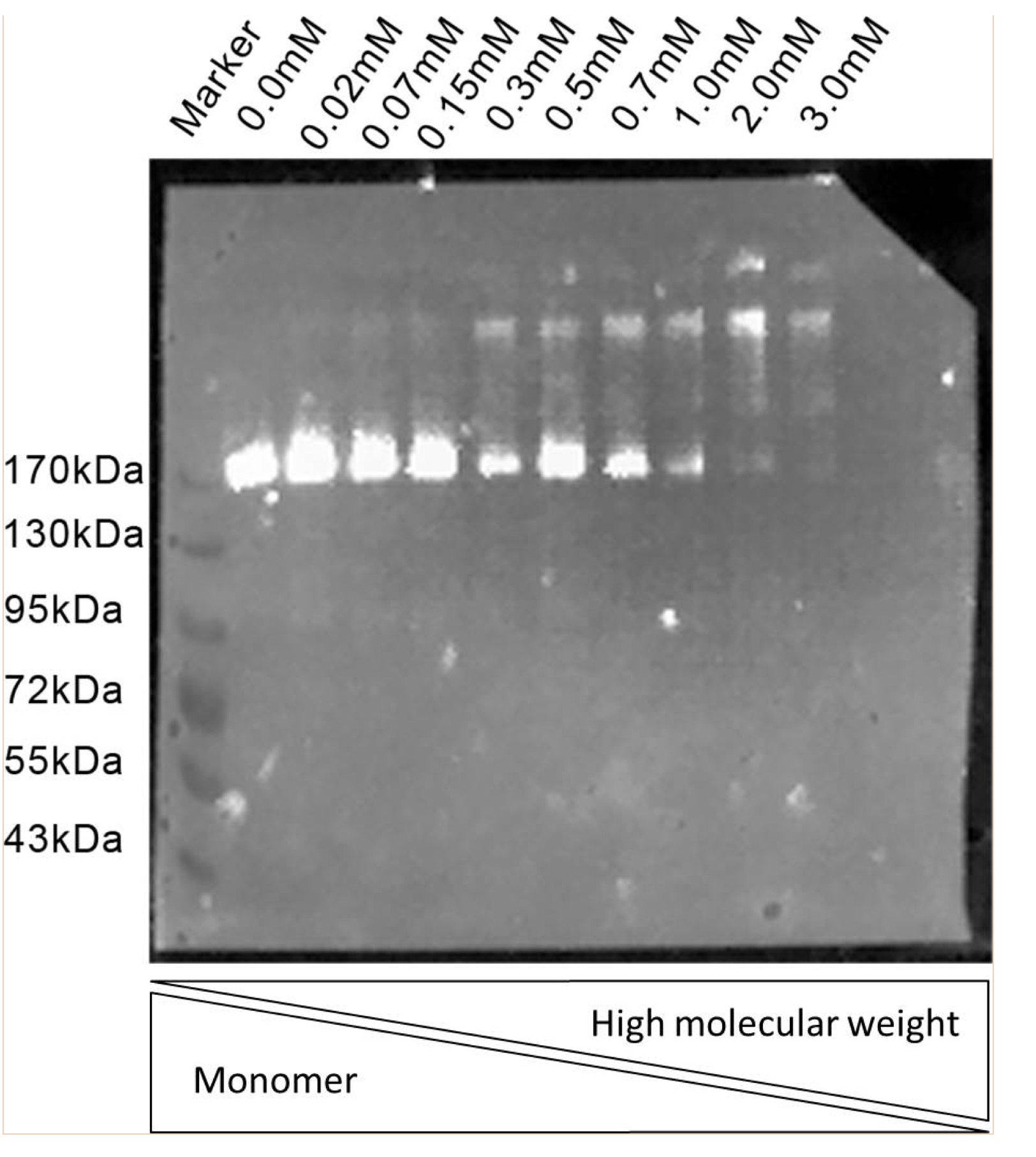

Supplement: S8 Fig — Immuno-detection (Western blot) of the GlCHC-HA reporter presents a shift from the monomeric form to higher molecular weight complexes, with increasing concentrations of DSP (0–3 mM). Molecular size (kDa) marker bands are indicated on the left. (TIF) [file ppat.1005756.s010.tif]

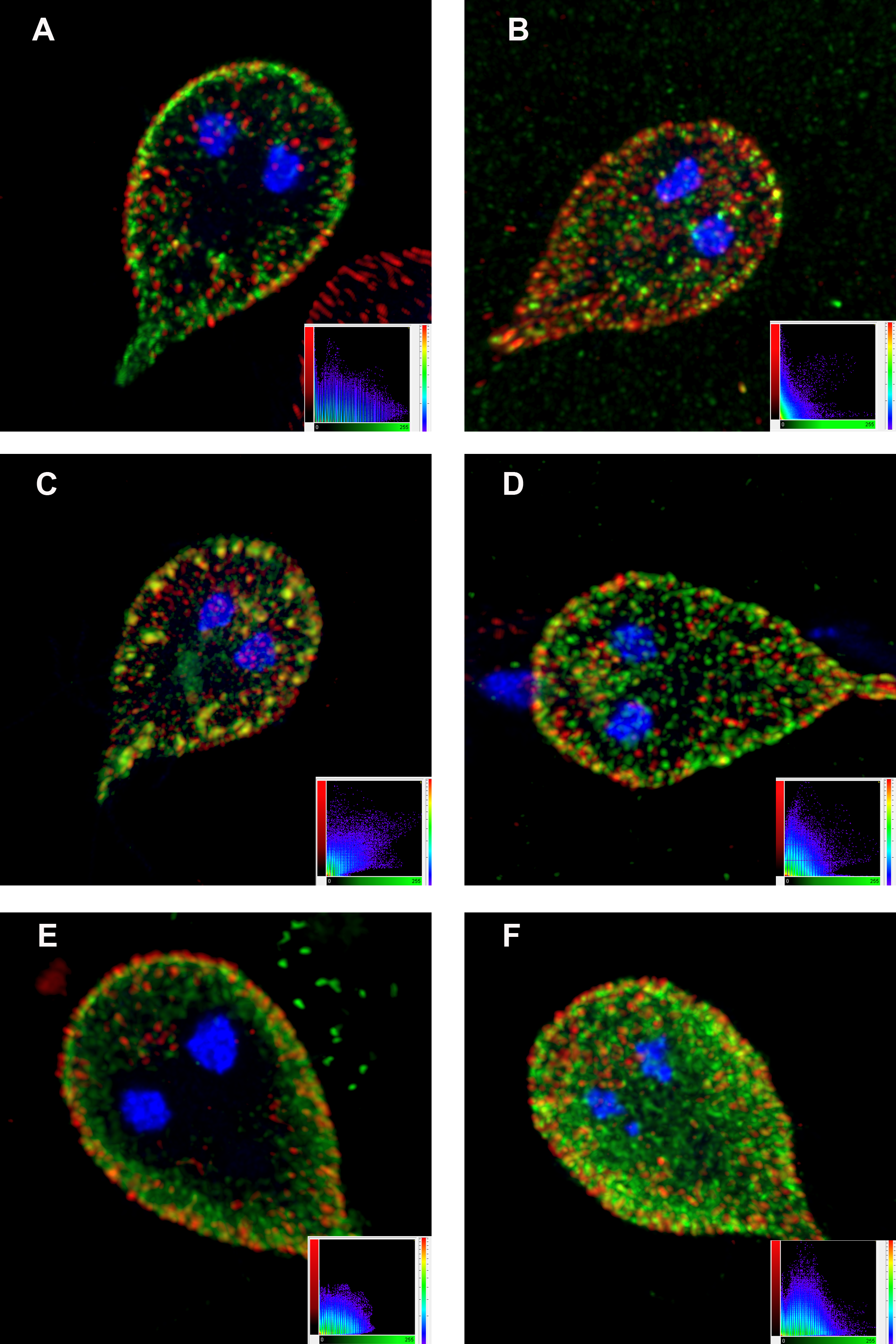

Supplement: S9 Fig — Immunofluorescence assays and confocal microscopy analysis of HA-tagged reporter lines for ORFs 15411, 7723, 16595, 16653, 10358, and 6687 (A–F), labeled for both the HA tag (green) and endogenous GlCHC (red). Insets: two dimensional scatter plots showing signal overlap (green, red) in voxels. Nuclei are labelled with DAPI (blue). (TIF) [file ppat.1005756.s011.tif]

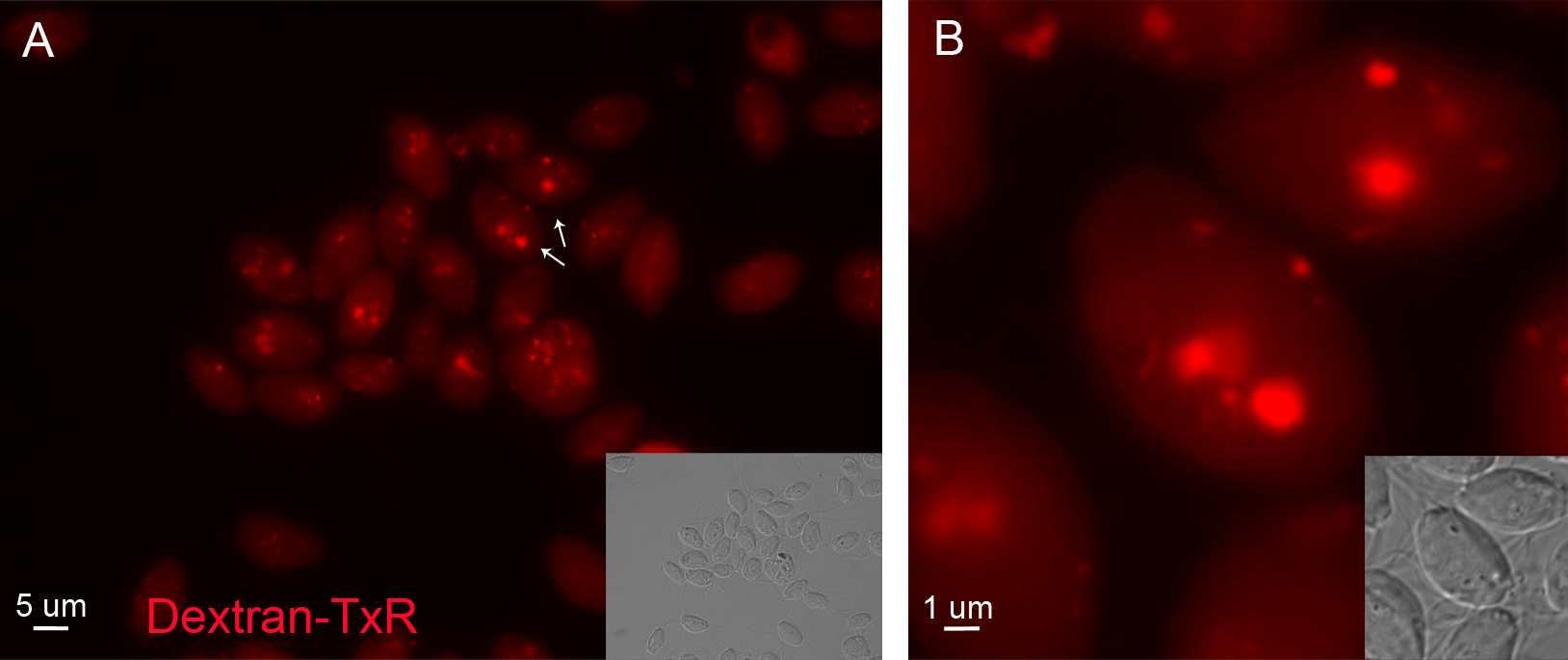

Supplement: S10 Fig — (A) The fluid phase marker dextran-TxR is taken up into intracellular compartments of varying sizes. (B) Inset of (A) as indicated by the arrows. Insets: DIC images. (TIF) [file ppat.1005756.s012.tif]

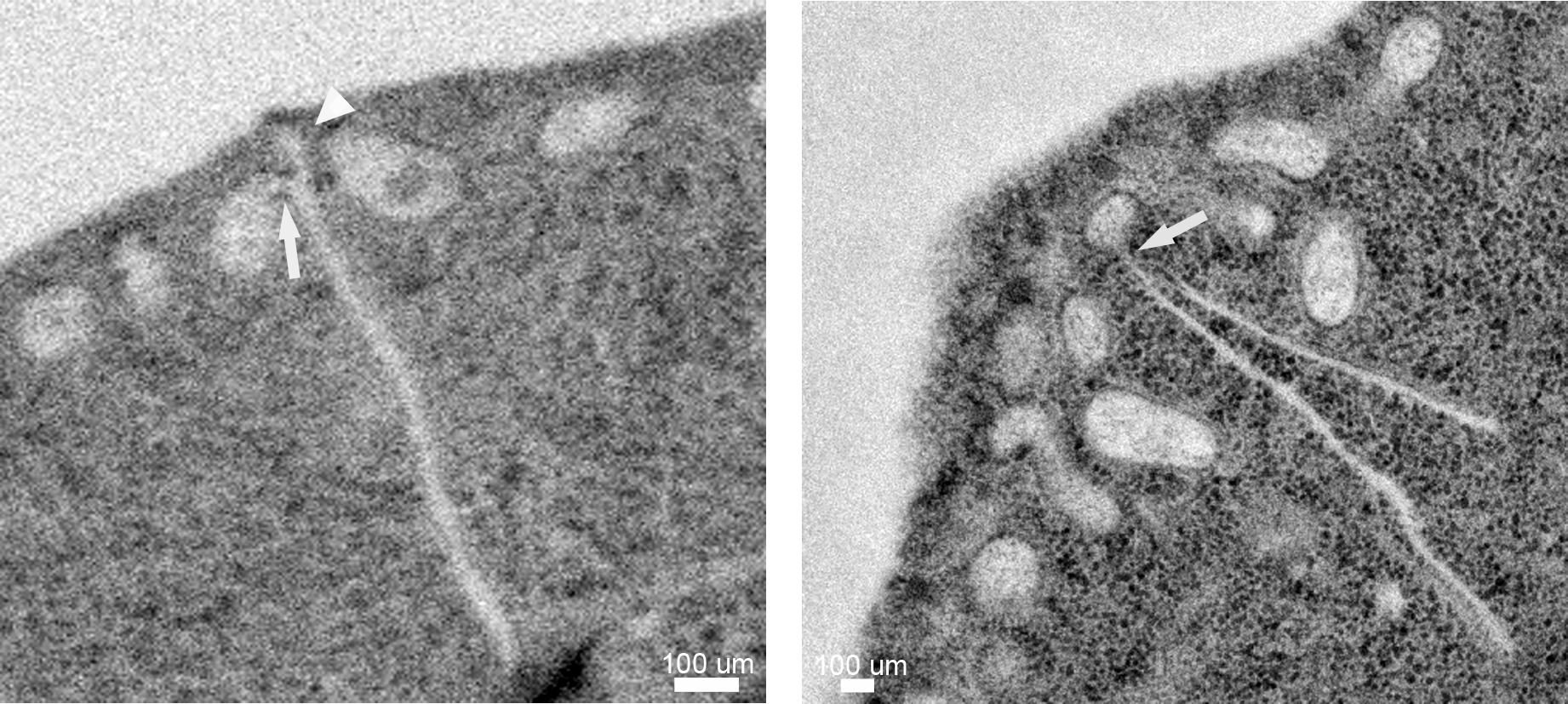

Supplement: S11 Fig — (TIF) [file ppat.1005756.s013.tif]
